# An Improved Approach for Practical Synthesis of 5-Hydroxymethyl-2′-deoxycytidine (5hmdC) Phosphoramidite and Triphosphate

**DOI:** 10.3390/molecules27030749

**Published:** 2022-01-24

**Authors:** Dong-Zhao Yang, Zhen-Zhen Chen, Mei Chi, Ying-Ying Dong, Shou-Zhi Pu, Qi Sun

**Affiliations:** 1 Jiangxi Key Laboratory of Organic Chemistry, Jiangxi Science and Technology Normal University, Nanchang 330013, China; yangdongzhao1@126.com (D.-Z.Y.); chenzhenzhen5@126.com (Z.-Z.C.); chimei5@126.com (M.C.); dongyingying0@126.com (Y.-Y.D.); 2 Department of Ecology and Environment, Yuzhang Normal University, Nanchang 330103, China

**Keywords:** 5-hydroxymethyl-2′-deoxycytidine (5hmdC), cyanoethyl ether, phosphoramidite, triphosphate, P(V)-N activation

## Abstract

5-Hydroxymethyl-2′-deoxycytidine (5hmdC) phosphoramidite and triphosphate are important building blocks in 5hmdC-containing DNA synthesis for epigenetic studies. However, efficient and practical methods for the synthesis of these compounds are still limited. The current research provides an intensively improved synthetic method that enables the preparation of commercially available cyanoethyl-protected 5hmdC phosphoramidite with an overall yield of 39% on 5 g scale. On the basis of facile and efficient accesses to cyanoethyl protected-5hmdU and 5hmdC intermediates, two efficient synthetic routes for 5hmdC triphosphate were also developed.

## 1. Introduction

5-Hydroxymethyl-2′-deoxycytidine (5hmdC) is a key oxidative intermediate in the demethylation pathway of 5-methyl-2′-deoxycytidine (5mdC) mediated by ten-eleven translocation (TET) proteins in epigenetic regulation [1,2,3,4,5,6,7]. More recently, 5hmdC has also been found to play pivotal roles in many crucial physiological processes, such as embryonic development [8], neurodevelopment [9,10,11], cellular differentiation [12], and initiation/progression of cancer [13,14]. However, it is believed that more biological functions of 5hmdC are still waiting to be unraveled [15,16]. To this end, synthesis of 5hmdC-containing oligodeoxynucleotides (ODNs) has been employed as an essential tool. Other than conventional phosphoramidite-based DNA synthesis with protected 5hmdC phosphoramidite as the building block [17,18], polymerase chain reaction (PCR) has also been utilized as an alternative approach to incorporate 5hmdC into DNA fragments by using 5hmdC triphosphate as the substrate [19,20]. 

**Figure 1:**
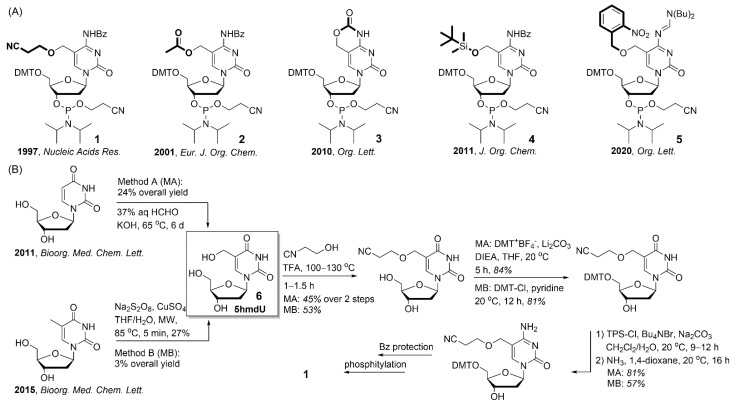
5hmdC Phosphoramidites with different protecting groups (**A**) and two improved synthetic methods for 5hmdC phosphoramidite **1** (**B**).

As shown in Figure 1A, five different types of 5hmdC phosphoramidite building blocks have been reported to date. 2-Cyanoethyl (**1**) is the first protecting group utilized for 5-CH_2_OH of 5hmdC [21]. Afterwards, acetyl (**2**) [22], cyclic carbamate (**3**) [23], and *t*-butyldimethylsilyl (**4**) [24] have also been employed as protecting groups. More recently, 2-nitrobenzyl-protected 5hmdC phosphoramidite (**5**) has enabled photocaged epigenetic labeling of DNA fragments at specific positions [25]. Currently, cyanoethyl-protected 5hmdC phosphoramidite (**1**) is most widely used for automated DNA synthesis and available from commercial suppliers. Though the final cleavage of cyanoethyl ether requires elongated reaction time and elevated temperature, however, its excellent stability during DNA synthesis, compatibility with existing DNA synthetic protocols, and clean deprotection are its advantages over other competitors. 

In 2011 and 2015, Schofield [26] and Shao [27] reported two improved methods for the synthesis of cyanoethyl-protected 5hmdC phosphoramidite (**1**) starting from 2’-deoxyuridine (dU) and thymidine (dT), respectively (Figure 1B). Other than the classic benzoyl (Bz) protection and phosphitylation steps, most of key steps including the initial conversion to 5-hydroxymethyl-2’-deoxyuridine (5hmdU), cyanoethyl protection, DMT protection, and *C*^4^-amination steps still suffer from either low-yielding or slow reaction rate. While Schofield’s route (Method A) gave **1** in 24% overall yield, Shao’s approach (Method B) afforded **1** in only 3% overall yield. Herein, we report an intensively optimized method for practical synthesis of **1** with an overall yield of 39% on 5 g scale. Moreover, two synthetic routes based on cyanoethyl protected-5hmdU and 5hmdC intermediates were also developed to access 5hmdC triphosphate on 50 mg scale. 

## 2. Results and Discussion

### 2.1. Synthesis of Cyanoethyl-Protected 5hmdC Phosphoramidite (***1***)

**Scheme 1:**
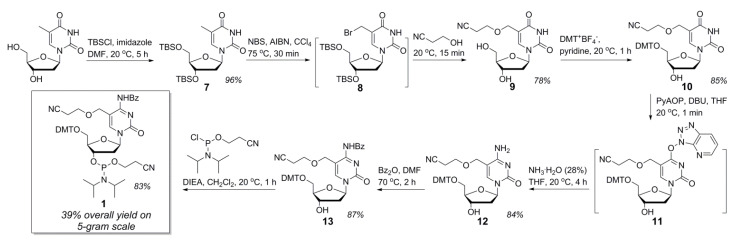
An improved method for practical synthesis of cyanoethyl-protected 5hmdC phosphoramidite (**1**).

As illustrated in Scheme 1, our method employed inexpensive dT starting material, which was first converted to diTBS-protected dT (**7**) in 96% yield and purified via simple precipitation. After free radical bromination at 5-methyl position, 5-bromomethyl dU (5BrmdU) intermediate (**8**) was concentrated and directly treated with 2-cyanoethanol at ambient temperature for 15 min to afford cyanoethyl-protected 5hmdU (**9**) in 78% yield. Treating **9** with DMT tetrafluoroborate (DMT^+^BF_4_^−^) in pyridine resulted in fast and efficient introduction of DMT to the sterically hindered 5’-OH (85%, **10**). According to the new *C*^4^-amination method we developed, instant activation with PyAOP (1 min, **11**) followed by aminolysis with conc. ammonia (4 h) yielded 5hmdC intermediate (**12**) in 84% yield. *N*-Benzoylation of **12** with benzoic anhydride in DMF at 70 °C greatly shortened reaction time of **13** from 2–4 days (at ambient temperature) [26] to only 2 h without adverse effects on either yield or chemoselectivity. Finally, phosphitylation with 2-cyanoethyl *N*,*N*-diisopropylchlorophosphoramidite in the present of triethylamine afforded the desired cyanoethyl-protected 5hmdC (**1**) in 83% yield. Compared to Schofield’s and Shao’s methods, the current approach achieved 39% overall yield in 5 g scale practical synthesis.

**Scheme 2:**
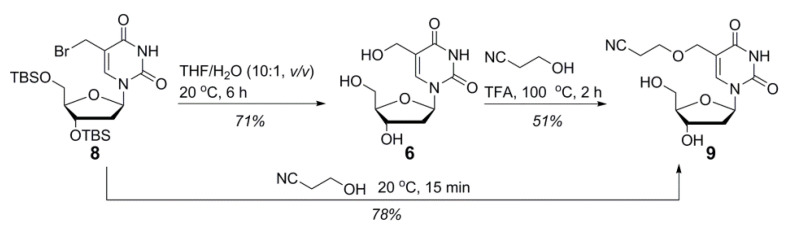
One-step synthesis of cyanoethyl-protected 5hmdU (**9**) from diTBS-protected 5BrhmdU (**8**).

Originally, we found that 5hmdU (**6**) could be efficiently obtained by hydrolyzing 5BrmdU (**8**) with TFA/H_2_O (10:1, *v*/*v*) in 71% yield. However, the following synthesis of cyanoethyl ether in the presence of TFA at 100 °C according to the previous reports [26,27] yielded the desired **9** in only 51% yield. Therefore, we abandoned the synthesis of 5hmdU and subsequent condensation with cyanoethanol under harsh conditions. Instead, we attempted to convert **8** directly to cyanoethyl ether **9** via alcoholysis with cyanoethanol under mild conditions. The simultaneous alcoholysis and cleavage of TBS by HBr generated in situ only took 15 min at ambient temperature, and column purification afforded **9** in 78% yield (Scheme 2).

In the following tritylation of 5’-OH with DMT-Cl in pyridine, we encountered the same incomplete reaction and low yielding scenario (51%, 12 h) mentioned by Schofield and coworkers [26] due to the elevated steric hindrance resulting from cyanoethyl protecting group. To solve this problem, we increased the reaction temperature to 40 and 60 °C (Table 1, Entry 1–3). However, the yield of **10** dropped ever lower due to the formation of more diDMT-protected side product. In addition, we also tested the reactions with a catalytic amount of DMAP, 2 equiv of DIPEA, and a new tritylation reagent, DMT-trifluoroacetate (Table 1, Entry 4–7) [28]. However, none of these conditions led to improved results. Surprisingly, when DMT^+^BF_4_^−^ along with additives was applied according to Schofield’s method, the yield of **10** was still very low (Table 1, Entry 8) [26]. Fortunately, our further optimization found that DMT^+^BF_4_^−^ alone in pyridine (without any additives) could afford the desired 5’-*O* tritylated product **10** in 85% yield within only 1 h. A possible mechanistic explanation for this result is because pyridine favors the dissociation of DMT^+^BF_4_^−^ into ionic form. Compared to the covalently linked DMT-Cl, DMT^+^ cation is more flattened and, therefore, much easier accessible to the 5’-OH of **9**. 

**Scheme 3:**
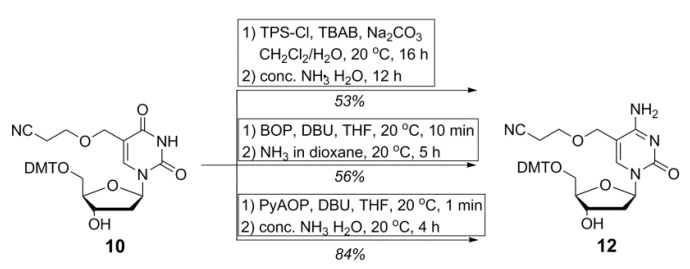
Comparison of TPS-Cl, BOP, and PyAOP-based amination methods for the synthesis of **12**.

The last bottle neck of the reported synthetic routes is the conversion of **10** to its dC counterpart **12**. The reported reaction yields based on 2,4,6-triisopropylbenzenesulfonyl chloride (TPS-Cl) activating reagent showed a large discrepancy (MA: 81% vs MB: 58%). In addition, both the biphasic activation and aminolysis steps were quite slow. In our hands, incomplete activation of urosine was observed and the yield of **11** (53%) was close to the one reported in Method B. We then tested the new BOP-based method reported by Lakshman and coworkers in 2017 [29]. However, the yield of **12** was still moderate (56%). To solve this problem, we systematically investigated the reactivity of different types of onium salt coupling reagents on the *C*^4^ amination and found that PyAOP is much more advantageous in both activation and aminolysis steps than BOP, especially in the case of structurally more hindered pyrimidine substrates such as **10**. Compared to the TPS-Cl method, the ultrafast activation by PyAOP (1 min) followed by aminolysis with concentrated ammonia (4 h) afforded **12** in 84% yield (Scheme 3). The more detailed condition optimization, mechanistic investigation, and application scope of onium salt-based *C*^4^-amination method for oxidized 5mdC derivatives have been described in a previous research article reported by our group [30]. However, it should be noted that, similar to the above mentioned DMT^+^BF_4_^−^, PyAOP is an onium salt coupling reagent. Therefore, the mechanistic rationale for its superb activating reactivity on sterically hindered **10** may also be ascribed to the significantly lowered steric hindrance of more flattened tripyrrolidinophosphonium ion.

### 2.2. Synthesis of 5hmdC Triphosphate (***14***)

As mentioned above, 5hmdC triphosphate (**14**) is the key substrate for PCR-based 5hmdC-containing DNA synthesis. Previously, we have reported a synthetic route for **14**, starting from acetyl/diTBS-protected 5hmdC [31]. The current synthetic advances made in the preparation of cyanoethyl/DMT-protected 5hmdC phosphoramidite (**1**) offered an excellent opportunity to gain more efficient accesses to **14**.

**Scheme 4:**
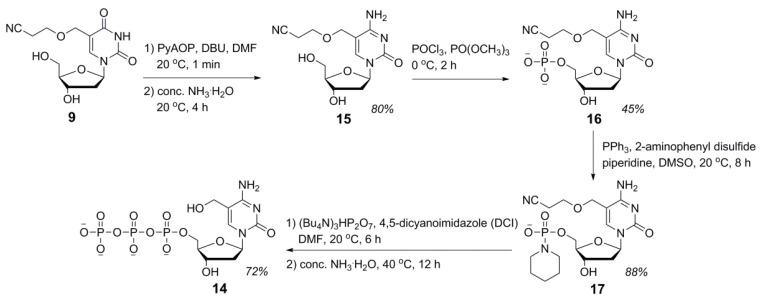
Cyanoethyl-protected 5hmdU (**9**)-based synthetic route for **14**.

In the first synthetic route (Scheme 4), we started with cyanoethyl-protected 5hmdU (**9**). Activation by PyAOP (1 min) followed by aminolysis with concentrated ammonia (4 h) yielded cyanoethyl-protected 5hmdC (**15**) in 80% yield. However, regioselective phosphorylation at 5′-OH position gave cyanoethyl-protected 5hmdC monophosphate (**16**) in only 45% yield possibly due to the increased steric hindrance resulting from the cyanoethyl ether. Redox condensation with PPh_3_, 2-aminophenyl disulfide, and piperidine smoothly mediated the conversion to phosphoropiperidate **17** (6 h, 88%) [32]. The coupling of **17** and pyrophosphate was conducted with 4,5-dicyanoimidazole (DCI) via the P(V)-N activation method. Then, the cyanoethyl protecting group was removed by treating the triphosphate with concentrated ammonia at 40 °C for 12 h. As has been reported [21,26], we found that concentrated ammonia can barely cleave cyanoethyl ether at ambient temperature. However, for small molecular substrates such as **14**, moderately elevated temperature (40 °C) is enough for its deprotection. The high temperature (75 °C) required in DNA synthesis is not necessary and impractical for triphosphate synthesis. Finally, purification by ion-exchange chromatography afforded the desired 5hmdC triphosphate (**14**) in 72% yield over two steps on 50 mg scale. Compared to our previous method based on Ac-protected 5hmdC intermediate (8 steps from diTBS-dT and 15% overall yield), the new route afforded **14** in 18% over 5 steps. 

**Scheme 5:**
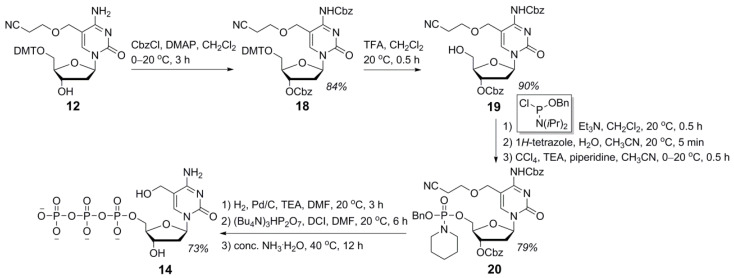
Fully protected phosphoropiperidate (**20**)-based synthetic route for **14**.

Due to the low-yielding monophosphorylation step in the first route, we chose to start with DMT/cyanoethyl-protected 5hmdC (**12**) in the second route and synthesize **14** via fully protected 5hmdC phosphoropiperidate intermediate (**20**) [33]. First, the 3’-OH and 4-NH_2_ of **14** were protected by benzyloxycarbonyl (Cbz) groups in 84% yield. After the DMT group was removed by TFA (90%, **19**), 5’-OH was phosphitylated with 2-benzyl *N*,*N*-diisopropylchlorophosphoramidite. In situ hydrolysis and oxidative coupling with piperidine yielded the fully protected phosphoropiperidate **20** in 79% yield over three consecutive steps. After Cbz and Bn protecting groups were removed by catalytic hydrogenation, the deprotected phosphoropiperidate **17** was treated with pyrophosphate in the presence of DCI as the promotor. The highly efficient P(V)-N activation method afforded the triphosphate in over 95% conversion as determined by ^31^P NMR. Finally, cleavage of cyanoethyl ether with concentrated ammonia at 40 °C followed by routine ion-exchange chromatography gave **14** in 73% yield over three consecutive steps on 50 mg scale (Scheme 5). Compared to the first synthetic route based on **9**, the second route based on **20** afforded **14** in a higher overall yield (24%) but required two extra steps (total 7 steps from diTBS-dT).

## 3. Materials and Methods

### 3.1. General Methods 

Chemical reagents and solvents were purchased from Leyan-Shanghai Haohong Scientific Co. Ltd., Shanghai, China. Reactions were monitored by TLC plates coated with 0.25 mm silica gel 60 F_254_ and visualized by UV irradiation (254 nm). Flash column chromatography employed silica gel (particle size 32–63 μm, Qingdao Haiyang Chemicals, Qingdao, China). NMR spectra were acquired on an AV-400 instrument (Bruker BioSpin, Faellanden, Switzerland) with chemical shifts reported in ppm and referenced to CDCl_3_, DMSO-*d_6_*, or D_2_O. IR spectra were obtained with a Vertex-70 instrument (Bruker Optics, Billerica, MA, USA). High-resolution mass spectra were obtained with a Dalton micrOTOF-Q II spectrometer (Bruker Optics, Billerica, MA, USA) and reported as *m*/*z*. Melting points were determined with an X-4 digital melting point apparatus and uncorrected (Tech Instrument, Beijing, China). The characterization data of **1** and **9**–**13** and NMR spectra of all synthesized compounds are provided in the Supplementary Materials. 

### 3.2. Synthetic Procedures of 5hmdC Phosphoramidite (***1***) and Intermediates (***9–13***)

5-(2-Cyanoethyl)hydroxymethyl-2′-deoxyuridine (**9**): To a solution of **7** (9.5 g, 20.3 mmol) in dry CCl_4_ (100 mL) were added NBS (5.78 g, 32.5 mmol) and AIBN (541 mg, 3.3 mmol). The reaction was stirred at 75 °C for 30 min until the orange color faded. Upon cooling, the reaction solution was filtered. The filtrate was concentrated in vacuo and dried under high vacuum for 30 min. The crude **8** was dissolved in cyanoethanol (6.9 mL, 101.5 mmol) and the reaction was stirred at 20 °C for 15 min. Then NaHCO_3_ (3.4 g, 40.6 mmol) was added to the solution to adjust pH to 7. The solution was washed with CH_2_Cl_2_ (200 mL × 5) to remove excess cyanoethanol. Flash column chromatography of the residual syrup (CH_2_Cl_2_/MeOH = 10:1) afforded **9** (4.9 g, 78%) as a white solid. 

5-(2-Cyanoethyl)hydroxymethyl-5′-(4,4′-dimethoxytrityl)-2′-deoxyuridine (**10**): To a solution of **9** (4.7 g, 15.2 mmol) in dry pyridine (60 mL) was added DMT^+^BF_4_^−^ (8.9 g, 22.8 mmol) under argon. The reaction was stirred at 20 °C for 1 h, then quenched with MeOH (5 mL), diluted with CH_2_Cl_2_ (150 mL), and washed with saturated aq. NaHCO_3_ solution (200 mL × 3) and H_2_O (200 mL). The organic layer was dried over anhydrous Na_2_SO_4_ and concentrated in vacuo. Flash column chromatography (CH_2_Cl_2_/MeOH = 20:1) afforded **10** (7.9 g, 85%) as a white solid.

5-(2-Cyanoethyl)hydroxymethyl-5′-(4,4′-dimethoxytrityl)-2′-deoxycytidine (**12**): To a solution of **10** (7.3 g, 11.9 mmol) and PyAOP (12.4 g, 23.8 mmol) in dry THF (60 mL) was added DBU (3.55 mL, 23.8 mmol) under argon at 0 °C. The reaction was stirred at 20 °C for 1 min and then concentrated NH_3_·H_2_O (28 w%, 7.2 mL, 95.2 mmol) was added. The reaction was stirred at 20 °C for 4 h and concentrated in vacuo. Flash column chromatography (DCM/MeOH = 10:1) afforded **12** (6.1 g, 84%) as a white solid.

*N*^4^-Benzoyl-5-(2-cyanoethyl)hydroxymethyl-5′-(4,4′-dimethoxytrityl)-2′-deoxycytidine (**13**): To a solution of **12** (6.0 g, 9.8 mmol) in dry THF (50 mL) was added benzoic anhydride (3.3 g, 14.7 mmol) under argon. The reaction was stirred at 70 °C for 2 h. Upon cooling, the reaction mixture was diluted with CH_2_Cl_2_ (150 mL). The solution was washed with saturated aq. NaHCO_3_ solution (200 mL), H_2_O (200 mL × 3). The combined organic layer was dried over anhydrous Na_2_SO_4_ and concentrated in vacuo. Flash column chromatography (PE/EA = 1:1) afforded **13** (6.1 g, 87%) as a white solid. 

*N*^4^-Benzoyl-5-(2-cyanoethyl)hydroxymethyl-5′-(4,4′-dimethoxytrityl)-2′-deoxycytidine-3′-[*N*,*N*-diisopropyl-*N*-cyanoethyl]phosphoramidite (**1**): To a solution of **13** (5.0 g, 7.0 mmol) in dry CH_2_Cl_2_ (50 mL) were added *N*,*N*-diisopropylethylamine (3.6 mL, 20.9 mmol) and 2-cyanoethyl *N*,*N*-diisopropylchlorophosphoramidite (3.1 mL, 14.0 mmol) under argon. The reaction was stirred at 20 °C for 1 h and then aq. NaHCO_3_ solution (5 w%, 100 mL) was added. The reaction mixture was extracted with CH_2_Cl_2_ (100 mL × 3). The organic layer was dried over anhydrous Na_2_SO_4_ and concentrated in vacuo. Flash column chromatography (PE/EA = 1:1, 0.5% TEA) afforded **1** (5.3 g, 83%) as a white solid. 

### 3.3. Synthetic Procedures and Characterization Data of 5hmdC Triphosphate (***14***) and Intermediates (***15–20***) 

3-((4-Amino-1-((2*R*,4*S*,5*R*)-4-hydroxy-5-(hydroxymethyl)tetrahydrofuran-2-yl)-2-oxo-1,2-dihydropyrimidin-5-yl)methoxy)propanenitrile (**15**): To a solution of **9** (930 mg, 3 mmol) in dry DMF (10 mL) was added PyAOP (2.5 g, 4.8 mmol) and DBU (730 mg, 4.8 mmol). The reaction was stirred at 20 °C for 1 min and then concentrated NH_3_·H_2_O (28 w%, 460 μL, 12 mmol) was added. The reaction was stirred at 20 °C for 4 h and then concentrated in vacuo. Flash column chromatography (CH_2_Cl_2_/MeOH = 8:1) afforded **15** (746 mg, 80%) as a white solid; mp 176–177 °C. ^1^H NMR (400 MHz, D_2_O): *δ* 7.93 (s, 1H), 6.16 (t, *J* = 6.4 Hz, 1H), 4.40–4.36 (m, 3H), 4.04–3.96 (m, 1H), 3.85–3.77 (m, 1H), 3.76–3.72 (m, 1H), 3.71–3.66 (m, 2H), 2.75 (t, *J* = 5.9 Hz, 2H), 2.45–2.36 (m, 1H), 2.28–2.19 (m, 1H) ppm; ^13^C NMR (100 MHz, D_2_O): *δ* 164.6, 156.6, 142.1, 120.0, 103.5, 86.9, 86.4, 70.4, 65.6, 64.3, 61.2, 39.7, 18.3 ppm; IR (KBr): *v*_max_ 3470, 2900, 2267, 1666, 1489, 1304, 1093 cm^−1^; HRMS (ESI+) *m*/*z* calcd for C_13_H_18_N_4_O_5_Na [M + Na]^+^ 333.1169; found 333.1170. 

((2*R*,3*S*,5*R*)-5-(4-Amino-5-((2-cyanoethoxy)methyl)-2-oxopyrimidin-1(2*H*)-yl)-3-hydroxytetrahydrofuran-2-yl)methyl phosphate (**16**): To a solution of **15** (500 mg, 1.6 mmol) in dry PO(OCH_3_)_3_ (10 mL) was added POCl_3_ (489 mg, 3.2 mmol). The reaction was stirred at 0 °C for 2 h and concentrated in vacuo. The crude product was dissolved in deionized H_2_O (2 mL) and loaded on a DEAE Sephadex A-25 ion exchange column (2.6 × 20 cm). Elution with NH_4_HCO_3_ buffer (linear gradient 0.1 to 0.5 M), combination of appropriate fractions, and lyophilization afforded **16** (293 mg, 45%) in ammonium salt form as a white solid. ^1^H NMR (400 MHz, D_2_O): *δ* 8.05 (s, 1H), 6.26 (t, *J* = 6.6 Hz, 1H), 4.53–4.47 (m, 1H), 4.46 (s, 2H), 4.19–4.13 (m, 1H), 4.11–3.88 (m,2H), 3.70 (t, *J* = 5.9 Hz, 2H), 3.15 (q, *J* = 7.3 Hz, 1H), 2.74 (t, *J* = 5.8 Hz, 2H), 2.44–2.35 (m, 1H), 2.29–2.21(m, 1H) ppm; ^13^C NMR (100 MHz, D_2_O): *δ* 164.8, 156.7, 142.2, 120.0, 103.8, 86.4, 86.0, 85.9, 71.0, 66.6, 64.6, 64.1, 46.9, 39.9, 18.4, 8.4 ppm; ^31^P NMR (162 MHz, D_2_O): *δ* −0.08 ppm; IR (KBr): *v*_max_ 3345, 2982, 2854, 2260, 1694, 1671, 1415, 1226, 1060, 928, 852 cm^−1^; HRMS (ESI-) *m*/*z* calcd for C_13_H_18_N_4_O_8_P [M − H]^−^ 389.0868; found 389.0868. 

((2*R*,3*S*,5*R*)-5-(4-Amino-5-((2-cyanoethoxy)methyl)-2-oxopyrimidin-1(2*H*)-yl)-3-hydroxytetrahydrofuran-2-yl)methyl piperidin-1-ylphosphonate (**17**): To a solution of **16** (220 mg, 0.45 mmol, triethylammonium form) and piperidine (192 mg, 2.25 mmol) in DMSO (4 mL) were added 2-aminophenyl disulfide (335 mg, 1.35 mmol) and triphenylphosphine (655 mg, 2.5 mmol). The reaction was stirred at 20 °C for 8 h. Then, a solution of NaI in acetone (0.5 M, 5 mL) was added dropwise. The resulting white precipitated sodium salt was collected by centrifuge. Passage of the solution of the sodium salt in deionized H_2_O through a bed of Dowex 50W-X8 ion exchange resin (Et_3_NH^+^ form) and lyophilization afforded **17** (240 mg, 88%) in triethylammonium salt form as white hydroscopic foam. ^1^H NMR (400 MHz, D_2_O): *δ* 8.00 (s, 1H), 6.26 (t, *J* = 6.5 Hz, 1H), 4.54–4.51 (m, 1H), 4.49 (s, 2H), 4.20–4.15 (m, 1H), 4.02–3.91 (m, 2H), 3.75 (t, *J* = 5.6 Hz, 2H), 3.18 (q, *J* = 7.2 Hz, 9H), 2.94–2.88 (m, 4H), 2.78 (t, *J* = 5.2 Hz, 2H), 2.48–2.41 (m, 1H), 2.35–2.23 (m, 1H), 1.46–1.41(m, 6H), 1.26 (t, *J* = 7.2 Hz, 14H) ppm; ^13^C NMR (100 MHz, D_2_O): *δ* 165.2, 157.3, 141.9, 119.9, 103.7, 86.5, 86.2, 71.2, 66.8, 64.3, 64.1, 46.8, 45.9, 40.0, 25.8, 24.1, 18.3, 8.3 ppm; ^31^P NMR (162 MHz, D_2_O): δ 9.23 ppm; IR (KBr): *v*_max_ 3458, 2262, 1670, 1562, 1420, 1333, 1126, 1058, 967, 854 cm^−1^; HRMS (ESI-) *m*/*z* calcd for C_18_H_27_N_5_O_7_P [M − H]^−^ 456.1654; found 456.1653. 

Benzyl(1-((2*R*,4*S*,5*R*)-4-(((benzyloxy)carbonyl)oxy)-5-((bis(4-methoxyphenyl)(phenyl)methoxy)methyl)tetrahydrofuran-2-yl)-5-((2-cyanoethoxy)methyl)-2-oxo-1,2-dihydropyrimidin-4-yl)carbamate (**18**): To a solution of **12** (500 mg, 0.82 mmol) and DMAP (300 mg, 2.46 mmol) in dry CH_2_Cl_2_ (8 mL) was added Cbz-Cl (253 μL, 1.80 mmol) at 0 °C. The reaction was slowly warmed to ambient temperature, stirred at 20 °C for 3 h, and concentrated in vacuo. Flash column chromatography (PE/EA = 8:1) afforded **18** (604 mg, 84%) as a white solid; mp 92–93 °C. ^1^H NMR (400 MHz, CDCl_3_): *δ* 12.21 (br, 1H), 7.89 (s, 1H), 7.42–7.24 (m, 19H), 6.86–6.81 (m, 4H), 6.40–6.34 (m, 1H), 5.29–5.24 (m, 1H), 5.19 (s, 2H), 5.14 (s, 2H), 4.20 (s, 1H), 4.09–4.04 (m, 1H), 3.92–3.87 (m, 1H), 3.79 (s, 6H), 3.57–3.53 (m, 1H), 3.34–3.31 (m, 1H), 3.28 (t, *J* = 6.9 Hz, 2H), 2.62–2.55 (m, 1H), 2.43–2.34 (m, 1H), 1.96 (t, *J* = 6.9 Hz, 2H) ppm; ^13^C NMR (100 MHz, CDCl_3_): *δ* 163.8, 159.3, 159.0, 154.4, 147.6, 144.5, 137.8, 136.2, 135.6, 135.4, 134.9, 130.3, 130.2, 128.9, 128.6, 128.5, 128.4, 128.3, 127.3, 117.5, 113.5, 111.9, 87.1, 85.1, 84.0, 78.4, 77.5, 76.8, 70.2, 68.0, 65.6, 65.3, 63.6, 55.4, 38.5, 17.9 ppm; IR (KBr): *v*_max_ 3476, 3263, 2934, 2837, 2263, 1732, 1654, 1610, 1560, 1504, 1415, 1394, 1326, 1283, 1249, 1195, 1098, 1059, 1026, 981, 829, 741 cm^−1^; HRMS (ESI+) *m*/*z* calcd for C_50_H_48_N_4_O_11_Na [M + Na]^+^ 903.3212; found 903.3210.

Benzyl(1-((2*R*,4*S*,5*R*)-4-(((benzyloxy)carbonyl)oxy)-5-(hydroxymethyl)tetrahydrofuran-2-yl)-5-((2-cyanoethoxy)methyl)-2-oxo-1,2-dihydropyrimidin-4-yl)carbamate (**19**): The solution of **18** (500 mg, 0.57 mmol) in CH_2_Cl_2_/TFA (*v*/*v* = 50:1, 10 mL) was stirred at 20 °C for 30 min and concentrated in vacuo. Flash column chromatography (PE/EA = 2:1) afforded **19** (297 mg, 90%) as a white solid; mp 136–138 °C. ^1^H NMR (400 MHz, CDCl_3_): *δ* 12.20 (br, 1H), 8.10 (s,1H), 7.43–7.28 (m, 10H), 6.36–6.31 (m, 1H), 5.30 (d, *J* = 5.9 Hz, 1H), 5.18–5.16 (m, 4H), 4.43 (s, 2H), 4.24–4.22 (m, 1H), 3.96 (d, *J* = 11.1 Hz, 1H), 3.87 (d, *J* = 11.5 Hz, 1H), 3.75 (t, *J* = 5.8 Hz, 2H), 2.98 (s, 1H), 2.68 (t, *J* = 5.8 Hz, 2H), 2.56–2.50 (m, 1H), 2.42–2.33 (m, 1H) ppm; ^13^C NMR (100 MHz, CDCl_3_): *δ* 163.6, 159.0, 154.6, 147.7, 137.6, 136.2, 134.9, 128.9, 128.8, 128.7, 128.6, 128.5, 128.4, 119.0, 111.3, 86.4, 85.3, 78.9, 70.2, 67.9, 65.9, 65.2, 62.5, 38.3, 19.3 ppm; IR (KBr): *v*_max_ 3265, 2914, 2266, 1723, 1650, 1613, 1565, 1512, 1415, 1392, 1326, 1285, 1194, 1095, 1056, 983, 740 cm^−1^; HRMS (ESI+) *m*/*z* calcd for C_29_H_30_N_4_O_9_Na [M + Na]^+^ 601.1905; found 601.1910.

Benzyl(1-((2*R*,4*S*,5*R*)-5-((((benzyloxy)(piperidin-1-yl)phosphoryl)oxy)methyl)-4-(((benzyloxy)carbonyl)oxy)tetrahydrofuran-2-yl)-5-((2-cyanoethoxy)methyl)-2-oxo-1,2-dihydropyrimidin-4-yl)carbamate (**20**): To a solution of **19** (250 mg, 0.44 mmol) and Et_3_N (180 μL, 1.3 mmol) in CH_2_Cl_2_ (10 mL) was added the solution of 2-benzyl *N*,*N*-diisopropylchlorophosphoramidite (205 mg, 0.75 mmol) in CH_2_Cl_2_ (3 mL). The reaction was stirred at 20 °C for 30 min and concentrated in vacuo. The residue was concentrated and coevaporated with CH_3_CN (10 mL × 2) and then dissolved in EtOAc (5 mL). The solution was filtered and concentrated in vacuo to afford the crude phosphoramidite. To a solution of the crude intermediate (ca. 0.44 mmol) in CH_3_CN (10 mL) was added 1H-tetrazole (61 mg, 0.87 mmol) and deionized H_2_O (200 μL). The reaction was stirred at 20 °C for 5 min and then concentrated in vacuo. The residue was dissolved in CH_2_Cl_2_ (20 mL) and washed with aq. HCl solution (0.02 M, 20 mL) and H_2_O (20 mL). The organic layer was dried over anhydrous Na_2_SO_4_ and concentrated in vacuo to afford the crude *H*-phosphonate intermediate. To a solution of the crude *H*-phosphonate in CH_3_CN (10 mL) were added piperidine (52 μL, 0.52 mmol), Et_3_N (300 μL), and CCl_4_ (220 μL) at 0 °C. The reaction was warmed to ambient temperature, stirred at 20 °C for 30 min, and concentrated in vacuo. The residue was dissolved in EtOAc (5 mL), filtered, and concentrated in vacuo. Flash column chromatography (PE/EA = 4:1) afforded **20** (277 mg, 79%) as a white solid, mp 44–45 °C. ^1^H NMR (400 MHz, CDCl_3_): *δ* 12.20 (br, 1H), 7.86–7.84 (m, 1H), 7.42–7.31 (m, 15H), 6.29–6.18 (m, 1H), 5.25–5.20 (m, 1H), 5.18–5.16 (m, 4H), 5.06–4.93 (m, 2H), 4.39–4.35 (m, 1H), 4.34–4.31 (m, 2H), 4.26–4.21 (m, 2H), 3.72–3.65 (m, 2H), 3.06–3.01 (m, 4H), 2.63–2.57 (m, 2H), 2.14–2.03 (m, 1H), 1.97 (s, 1H), 1.54–1.51 (m, 2H), 1.48–1.41 (m, 4H) ppm; ^13^C NMR (100 MHz, CDCl_3_): *δ* 163.7, 159.4, 159.3, 154.3, 147.5, 147.4, 138.2, 137.9, 136.3, 136.2, 136.1, 134.7, 128.9, 128.8, 128.7, 128.6, 128.5, 128.3, 127.9, 127.6, 118.0, 111.6, 111.4, 86.2, 85.8, 83.5, 78.1, 70.3, 68.2, 67.8, 65.6, 65.3, 45.4, 38.1, 26.1, 24.3, 18.7 ppm; ^31^P NMR (162 MHz, CDCl_3_): δ 8.77, 8.45 ppm; IR (KBr): *v*_max_ 3469, 3260, 2265, 1662, 1612, 1561, 1501, 1452, 1394, 1327, 1282, 1195, 1099, 979, 779, 741 cm^−1^; HRMS (ESI+) *m*/*z* calcd for C_41_H_46_N_5_O_11_PNa [M + Na]^+^ 838.2824; found 838.2820. 

5-Hydroxymethyl-2′-deoxycytidine 5′-triphosphate, tetrasodium salt (**14**): Method A: To a solution of **17** (67 mg, 0.11 mmol) in DMF (2 mL) were added tris(tetra-*n*-butyl ammonium) hydrogen pyrophosphate (198 mg, 0.22 mmol) and DCI (78 mg, 0.66 mmol). The reaction was stirred at 20 °C for 6 h and concentrated in vacuo. The residue was dissolved in NH_3_·H_2_O (28 w%, 5 mL), stirred at 40 °C for 12 h, and concentrated in vacuo. The residue was dissolved in aq. NaOAc solution (4 M, 0.5 mL) and then EtOH (10 mL) was added. The precipitated crude product was collected by filtration, dissolved in deionized H_2_O (0.5 mL), and loaded on a DEAE Sephadex A-25 ion exchange column (1.6 × 25 cm). Elution with NH_4_HCO_3_ buffer (linear gradient 0.2 to 0.6 M), combination of appropriate fractions, and lyophilization afforded **1** in ammonium salt form. Passage of the solution of the ammonium salt in deionized H_2_O through a bed of Dowex 50W-X8 ion exchange resin (Na^+^ form) and lyophilization afforded **1** (46 mg, 72%) as tetrasodium salt, a white solid. Method B: To a solution of **20** (106 mg, 0.13 mmol) and Et_3_N (16 μL, 0.13 mmol) in DMF (3.3 mL) was added 5 w% Pd/C (11 mg). The reaction was stirred at 20 °C under H_2_ atmosphere for 3 h. The catalyst was removed by a syringe filter (0.45 μm aperture) under argon. To the DMF solution was added tris(tetra-*n*-butylammonium) hydrogen pyrophosphate (242 mg, 0.26 mmol) and DCI (97 mg, 0.82 mmol). The reaction was stirred at 20 °C for 6 h and concentrated in vacuo. The residue was dissolved in NH_3_·H_2_O (28 w%, 5 mL) at 40 °C for 12 h and concentrated in vacuo. The residue was further purified according to the procedures described in Method A to afford **14** (55 mg, 73%) as tetrasodium salt, a white solid. ^1^H NMR (400 MHz, D_2_O): *δ* 7.94 (s, 1H), 6.24 (t, *J* = 6.4 Hz, 1H), 4.58 (m, 1H), 4.42 (s, 2H,), 4.17 (m, 3H), 2.37–2.25 (m, 2H) ppm; ^13^C NMR (100 MHz, D_2_O): *δ* 165.2, 157.4, 140.6, 107.1, 86.0, 85.7, 70.3, 65.1, 57.7, 39.5 ppm; ^31^P NMR (162 MHz, D_2_O): *δ* −7.90, −10.97, −22.05 ppm; IR: *v*_max_ 3346, 2986, 2856, 1694, 1413, 1226, 1080, 924, 813 cm^−1^; HRMS (ESI-): *m*/*z* calcd for C_10_H_18_N_3_O_14_P_3_ [M − H]^–^ 495.9711; found 495.9710. 

## 4. Conclusions

In summary, an improved method for practical synthesis of cyanoethtyl-protected 5hmdC phosphoramidite has been developed. Direct synthesis of cyanoethyl-protected 5hmdU via in situ alcoholysis and desilylation of 5BrmdU intermediate skipped the two limiting steps of the reported methods, i.e., the very low-yielding synthesis of 5hmdU and subsequent condensation with cyanoethanol under harsh conditions. In addition, the optimization of DMT and Bz protections along with application of the PyAOP-based *C*^4^ amination method also significantly increased the synthetic efficiency. In a 5 g synthesis, the overall yield of 5hmdC phosphoramidite was promoted from reported 24% to 39%. In addition, two practical routes for 5hmdC triphosphate synthesis on 50 mg scale have also been developed on the basis of easily accessible cyanoethyl protected-5hmdU and 5hmdC intermediates. Moreover, mechanistic consideration of the remarkably improved reactivity of tritylating and activating reagents (DMT^+^BF_4_^−^ and PyAOP) suggests that utilizing ionizable reagents could be an effective strategy to significantly promote the reactions on sterically hindered substrates due to their more flattened conformations.

## Figures and Tables

**Table 1:** Optimization of tritylation conditions for synthesis of **10**.

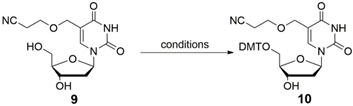		
Entry	Conditions	Yield (%)
1	DMT-Cl (1.5 eq), 20 °C, Pyr, 12 h	51 ^a^
2	DMT-Cl (1.5 eq), 40 °C, Pyr, 12 h	46 ^a^
3	DMT-Cl (1.5 eq), 60 °C, Pyr, 12 h	34 ^a,b^
4	DMT-Cl (1.5 eq), DMAP (0.15 eq), 20 °C, Pyr, 6 h	48 ^a^
5	DMT-Cl (1.5 eq), DMAP (0.15 eq), DIPEA (2 eq), 20 °C, Pyr, 6 h	54 ^a^
6	DMT-trifluoroacetate (1.5 eq), 20 °C, THF, 12 h	No reaction
7	DMT-trifluoroacetate (1.5 eq), 20 °C, Pyr, 12 h	No reaction
8	DMT^+^BF_4_^−^(1.5 eq), Li_2_CO_3_, (5 eq), DIPEA (3 eq), 20 °C, THF, 5 h	22 ^a^
9	DMT^+^BF_4_^−^ (1.5 eq), 20 °C, Pyr, 1 h	85

^a^**9** was not completely consumed when the reaction stopped. ^b^ diDMT-protected product formed.

## Data Availability

The data presented in this study are available in Supplementary Materials.

## Supplementary Materials

The following are available online, characterization data of **1** and **9**–**13** and Figures S1–S29: NMR spectra of all synthesized compounds.
