# Supplementary material for: An Improved Approach for Practical Synthesis of 5-Hydroxymethyl-2′-deoxycytidine (5hmdC) Phosphoramidite and Triphosphate
Source: Molecules. 2022 Jan 24;27(3):749. doi: 10.3390/molecules27030749 (PMC8839764; doi:10.3390/molecules27030749)
Supplement: Supplementary file 1 [file molecules-27-00749-s001.zip › molecules-1567293-supplementary.pdf]

Supporting Information

An improved approach for practical synthesis of 5-hydroxymethyl-2′-deoxycytidine (5hmdC) phosphoramidite and triphosphate

Dong-Zhao Yang[1], Zhen-Zhen Chen[1], Mei Chi[1], Ying-Ying Dong[1], Shou-Zhi Pu[1,2,*] and Qi Sun[1,*]

[1]*Jiangxi Key Laboratory of Organic Chemistry, Jiangxi Science and Technology Normal University, 605 Fenglin Avenue, Nanchang, Jiangxi 330013, PR China*

[2]*Department of Ecology and Environment, Yuzhang Normal University, Nanchang, Jiangxi 330103, PR China*

E-mail: pushouzhi@tsinghua.org.cn; sunqi@jxstnu.edu.cn

Table of contents

1. Characterization data of **1** and **9–13** Page S2–S3

2. ^{1}H, ^{13}C and ^{31}P NMR spectra of all synthesized compounds (Figure S1–S29) Page S4–S18

1. Characterization data of 1 and 9–13.

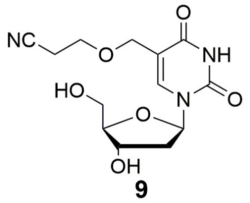

9

5-(2-Cyanoethyl)hydroxymethyl-2′-deoxyuridine (9): mp 79–81°C; ^{1}H NMR (400 MHz, DMSO-d_6): δ 11.37 (br, 1H), 7.87 (s, 1H), 6.08 (t, J = 6.7 Hz, 1H), 5.21 (d, J = 4.2 Hz, 1H), 4.98 (t, J = 5.1 Hz, 1H), 4.18–4.13 (m, 1H), 4.09 (d, J = 2.2 Hz, 2H), 3.73–3.68 (m, 1H), 3.55–3.46 (m, 4H), 2.67 (t, J = 6.0 Hz, 2H), 2.06–2.01 (m, 2H) ppm; ^{13}C NMR (100 MHz, DMSO-d_6): δ 162.9, 150.5, 139.6, 119.4, 110.2, 87.6, 84.4, 70.5, 64.8, 61.4, 48.8, 18.3 ppm; IR (KBr): ν_{max} 3423, 2944, 2260, 1683, 1207 cm^{-1}; LRMS (ESI+) *m/z* calcd for $C_{13}H_{18}N_3O_6$ [M+H]$^+$ 312.1; found 312.1.

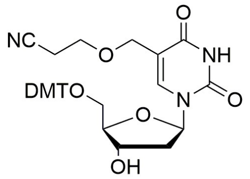

10

5-(2-Cyanoethyl)hydroxymethyl-5′-(4,4′-dimethoxytrityl)-2′-deoxyuridine (10): mp 87–88°C; ^{1}H NMR (400 MHz, CDCl$_3$): δ 9.52 (br, 1H), 7.84 (s, 1H), 7.40 (d, J = 7.4 Hz, 2H), 7.35–7.20 (m, 7H), 6.84 (d, J = 7.7 Hz, 4H), 6.39 (t, J = 7.2 Hz, 1H), 4.58–4.52 (m, 1H), 4.05–4.04 (m, 1H), 3.89 (d, J = 11.8 Hz, 1H), 3.79 (s, 6H), 3.70 (d, J = 11.8 Hz, 1H), 3.50–3.46 (m, 1H), 3.36–3.30 (m, 3H), 2.47–2.41 (m, 1H), 2.34–2.27 (m, 1H), 2.25–2.18 (m, 2H) ppm; ^{13}C NMR (100 MHz, CDCl$_3$): δ 162.9, 158.9, 150.4, 144.7, 139.1, 135.7, 135.6, 130.3, 128.3, 128.2, 127.3, 117.8, 113.5, 111.5, 87.0, 86.3 , 85.1, 65.4, 65.1, 63.6, 55.5, 41.2, 18.4 ppm; IR (KBr): ν_{max} 3448, 2932, 2252, 1688, 1607, 1509, 1251 cm^{-1}; LRMS (ESI+) *m/z* calcd for $C_{34}H_{36}N_3O_8$ [M+H]$^+$ 614.2; found 614.2.

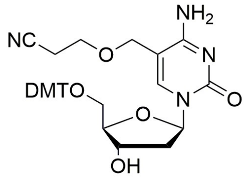

12

5-(2-Cyanoethyl)hydroxymethyl-5′-(4,4′-dimethoxytrityl)-2′-deoxycytidine (12): mp 143–145°C; ^{1}H NMR (400 MHz, CDCl$_3$): δ 7.93 (s, 1H), 7.39 (d, J = 7.4 Hz, 2H), 7.33–7.21 (m, 7H), 6.83 (d, J = 8.3 Hz, 4H), 6.51 (t, J = 6.5 Hz, 1H), 6.01 (br, 1H), 4.70 (br, 1H), 4.56–4.49 (m, 1H), 4.13 (d, J = 2.5 Hz, 1H), 3.78 (s, 6H), 3.73 (d, J = 12.4 Hz, 1H), 3.56–3.48 (m, 2H), 3.30–3.25 (m, 1H), 3.19–3.09 (m, 2H), 2.70–2.67 (m, 1H), 2.36–2.32 (m, 2H), 2.26–2.17 (m, 1H) ppm; ^{13}C NMR (100 MHz, CDCl$_3$): δ 165.2, 158.8, 156.2, 144.5, 140.7, 135.6, 130.3, 128.5, 128.1, 127.3, 117.6, 113.4, 102.2, 86.8, 86.6, 86.3, 72.1, 67.6, 64.0, 63.6, 55.4, 42.2, 18.5 ppm; IR (KBr): ν_{max} 3442, 2932, 2252, 1663, 1607, 1509, 1251 cm^{-1}; LRMS (ESI+) *m/z* calcd for $C_{34}H_{37}N_4O_7$ [M+H]$^+$ 613.3; found 613.3.

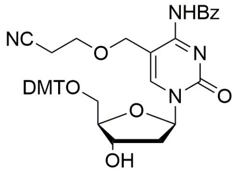

13

***N*[4]-Benzoyl-5-(2-cyanoethyl)hydroxymethyl-5′-(4,4′-dimethoxytrityl)-2′-deoxycytidine (13):** mp 89–90°C; ^{1}H NMR (400 MHz, $CDCl_3$): δ 13.3 (br, 1H), 8.23 (d, J = 7.3 Hz, 2H), 7.99 (s, 1H), 7.54–7.50 (m, 1H), 7.45–7.41 (m, 4H), 7.33–7.29 (m, 6H), 7.27–7.24 (m, 1H), 6.86–6.83 (m, 4H), 6.37 (t, J = 6.5 Hz, 1H), 4.56–4.53 (m, 1H), 4.27 (d, J = 12.1 Hz, 1H), 4.08–4.05 (m, 2H), 3.79 (s, 6H), 3.52 (dd, J_1 = 10.6 Hz, J_2 = 3.2 Hz, 1H), 3.44 (t, J = 6.6 Hz, 2H), 3.33 (dd, J_1 = 10.6 Hz, J_2 = 3.4 Hz, 1H), 2.52–2.43 (m, 1H), 2.36–2.32 (m, 1H), 2.16–2.11 (m, 2H) ppm; ^{13}C NMR (100 MHz, $CDCl_3$): δ 179.7, 159.0, 158.6, 147.0, 144.7, 140.7, 139.3, 137.0, 135.8, 135.7, 132.8, 130.4, 130.3, 130.0, 128.4, 128.2, 127.3, 117.7, 113.5, 111.9, 86.9, 86.3, 85.6, 72.0, 65.6, 63.5, 55.4, 41.4, 18.4 ppm; IR (KBr): ν_{max} 3439, 2932, 2252, 1709, 1648, 1604, 1568, 1251 cm^{-1}; LRMS (ESI+) *m/z* calcd for $C_{41}H_{41}N_4O_8$ $[M+H]^+$ 717.3; found 717.3.

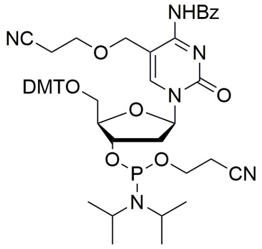

1

***N*[4]-Benzoyl-5-(2-cyanoethyl)hydroxymethyl-5′-(4,4′-dimethoxytrityl)-2′-deoxycytidine-3′-[*N*, *N*-diisopropyl-*N*-cyanoethyl]phosphoramidite (1).** mp 89–90°C; ^{1}H NMR (400 MHz, $CDCl_3$): δ 13.3 (br, 1H), 8.25 (d, J = 7.5 Hz, 2H), 8.06 (d, J = 15.3 Hz, 1H), 7.56–7.50 (m, 1H), 7.45–7.41 (m, 4H), 7.34–7.30 (m, 6H), 7.30–7.26 (m, 1H), 6.88–6.84 (m, 4H), 6.40 (dd, J_1 = 13.4 Hz, J_2 = 6.9 Hz, 1H), 4.66–4.63 (m, 1H), 4.24 (d, J = 12.0 Hz, 1H), 4.17 (d, J = 20.6 Hz, 1H), 3.95 (d, J = 11.9 Hz, 1H), 3.80 (s, 6H), 3.76 (m, 1H), 3.63–3.49 (m, 4H), 3.39–3.33 (m, 2H), 3.31–3.25 (m, 1H), 2.62 (t, J = 6.2 Hz, 1H), 2.59–2.52 (m, 1H), 2.42–2.32 (m, 2H), 2.07–1.98 (m, 2H), 1.19–1.14 (m, 9H), 1.03 (d, J = 6.7 Hz, 3H) ppm; ^{13}C NMR (100 MHz, $CDCl_3$): δ 179.6, 158.9, 158.5, 147.8, 144.5, 139.3, 139.2, 137.0, 135.6, 135.4, 132.7, 130.3, 130.2, 130.0, 128.4, 128.3, 128.1, 127.3, 127.2, 117.6, 113.4, 111.9, 86.7, 85.9, 85.6, 85.5, 85.4, 73.4, 73.2, 73.1, 73.0, 65.5, 63.0, 62.8, 58.5, 58.3, 58.2, 58.2, 55.4, 53.6, 43.5, 43.4, 43.3, 40.5, 24.7, 24.6, 24.5, 20.5, 20.4, 20.3, 20.2, 18.1 ppm; ^{31}P NMR (162 MHz, $CDCl_3$): δ 149.08, 148.61 ppm; LRMS (ESI+) *m/z* calcd for $C_{50}H_{58}N_6O_9P$ $[M+H]^+$ 917.4; found 917.4.

2. ^{1}H, ^{13}C and ^{31}P spectra of compounds

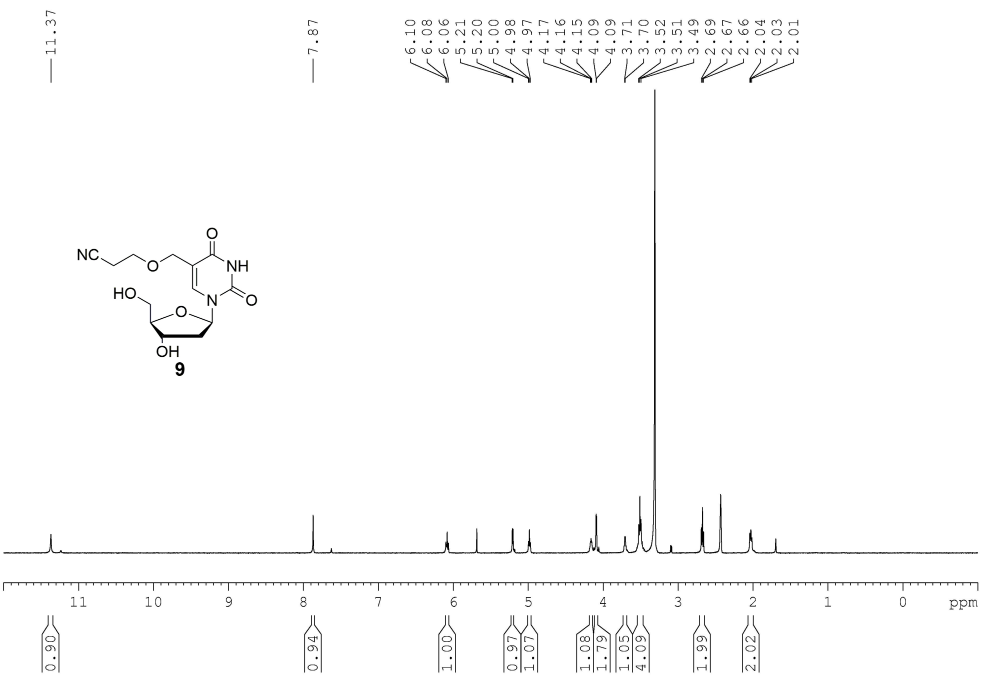

Figure S1. ^{1}H NMR spectrum of **9**

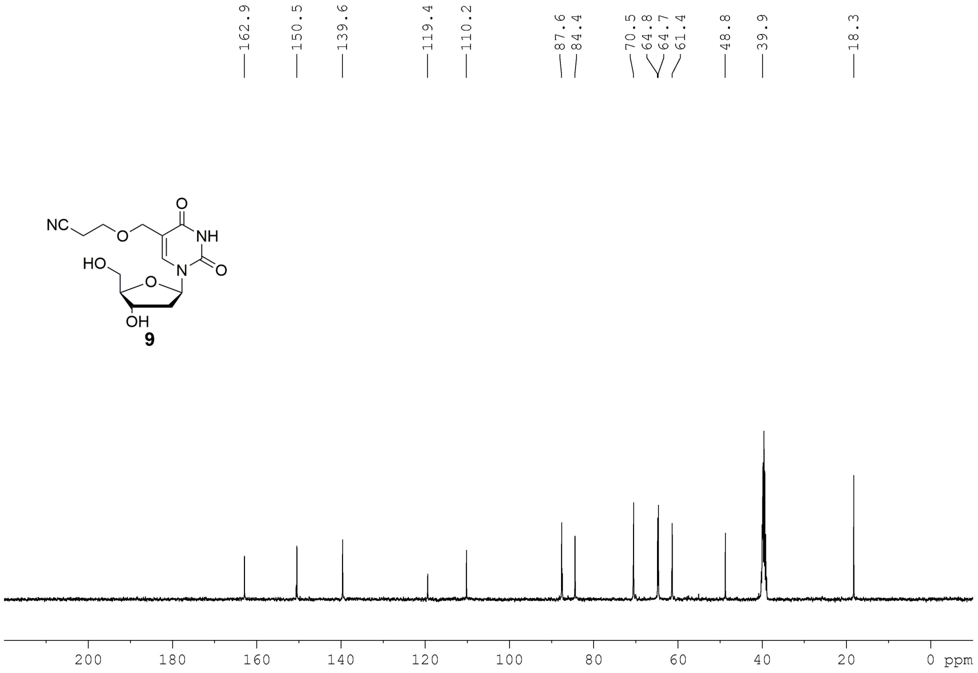

Figure S2. ^{13}C NMR spectrum of **9**

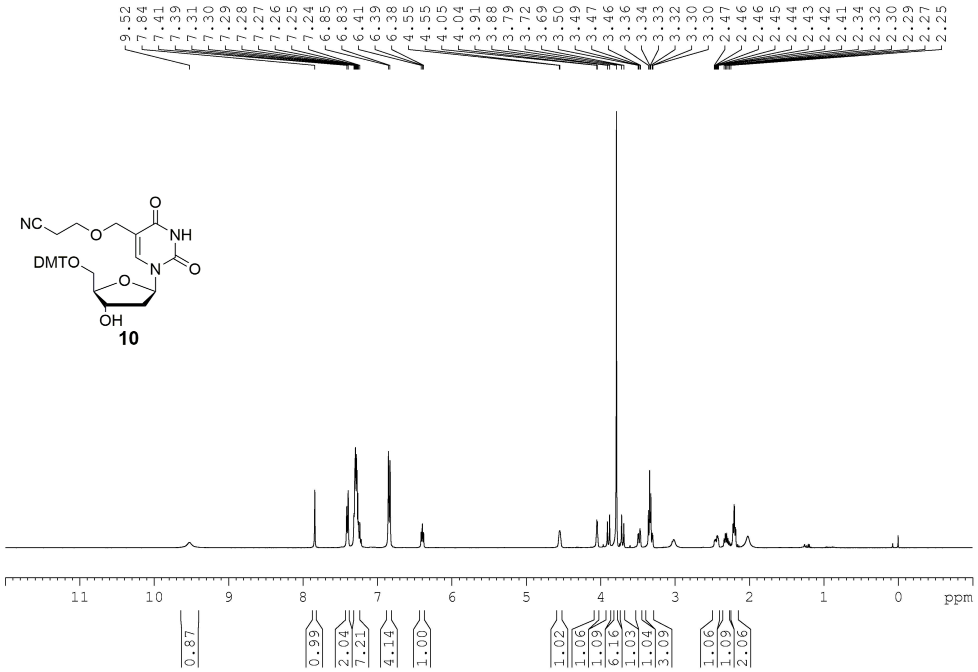

Figure S3. ^{1}H NMR spectrum of **10**

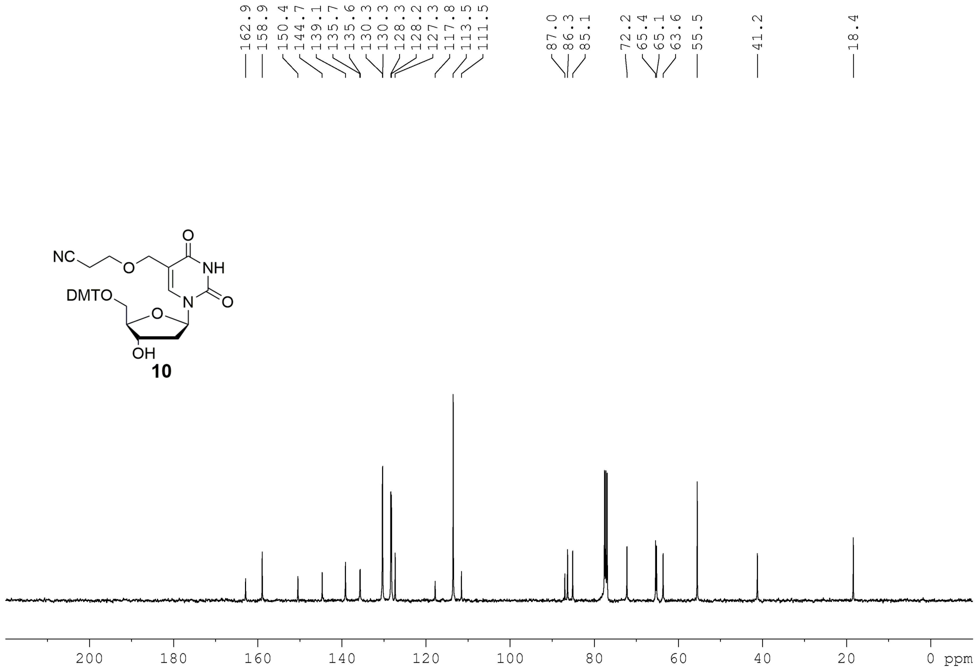

Figure S4. ^{13}C NMR spectrum of **10**

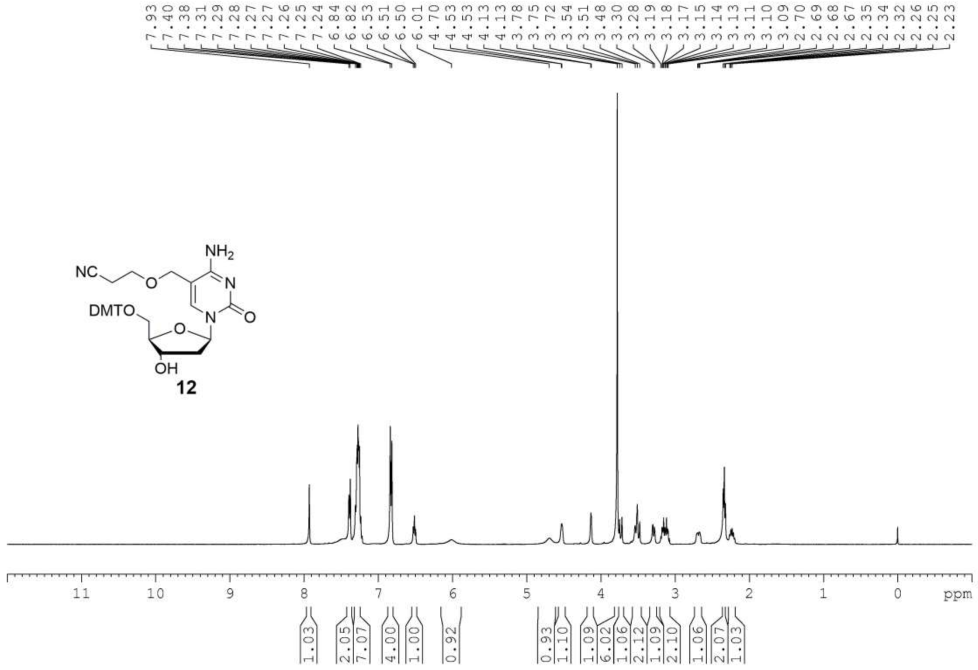

Figure S5. ^{1}H NMR spectrum of **12**

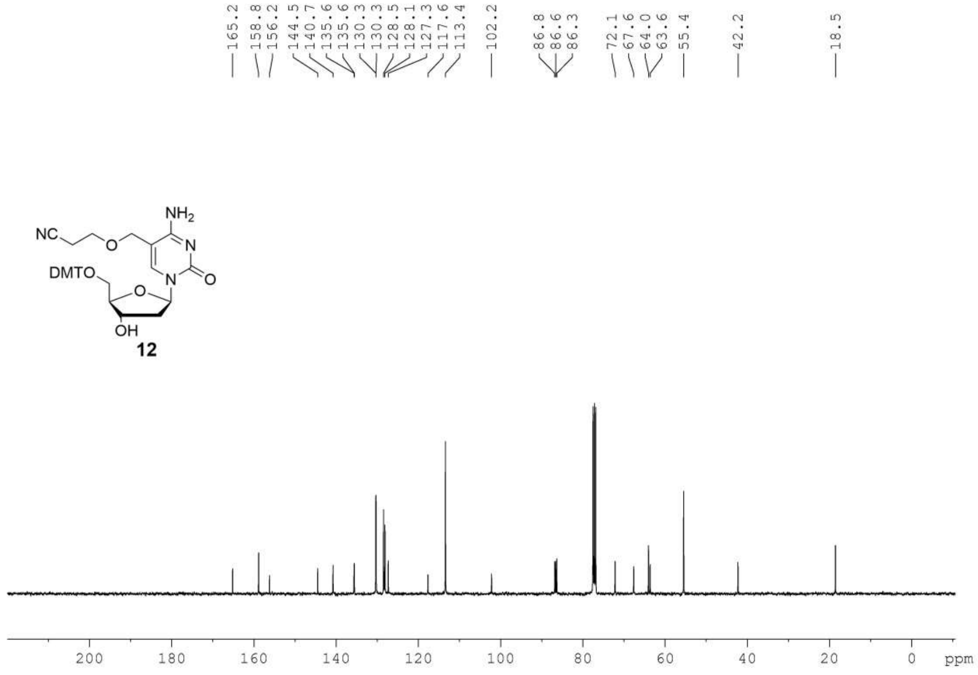

Figure S6. ^{13}C NMR spectrum of **12**

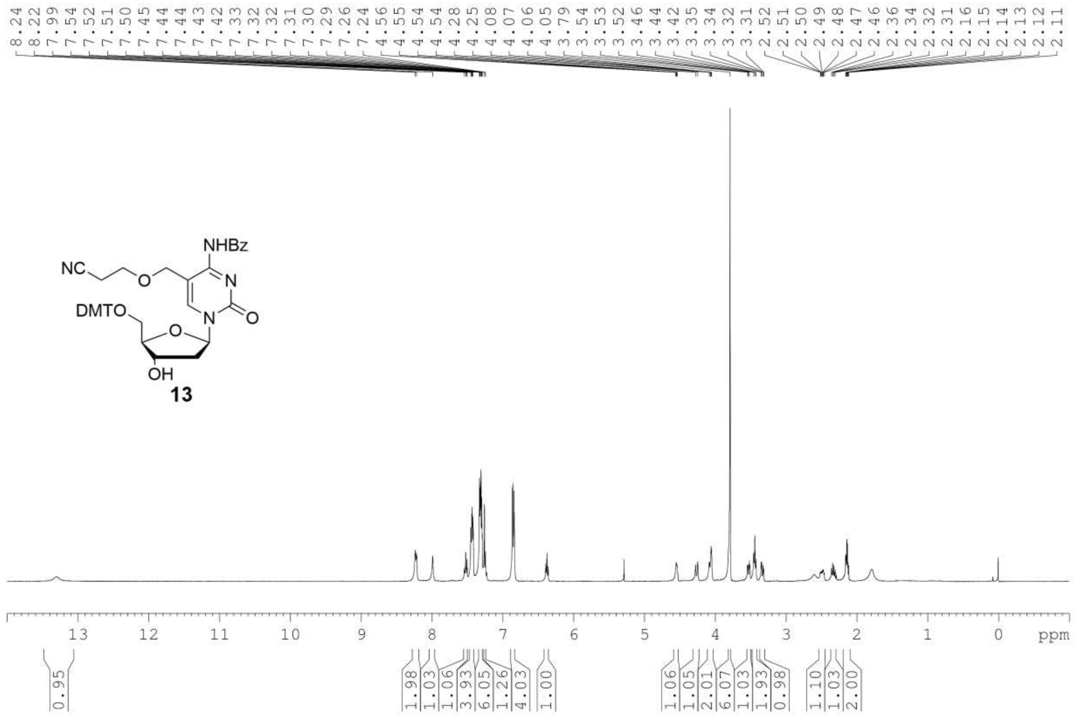

Figure S7. ^{1}H NMR spectrum of **13**

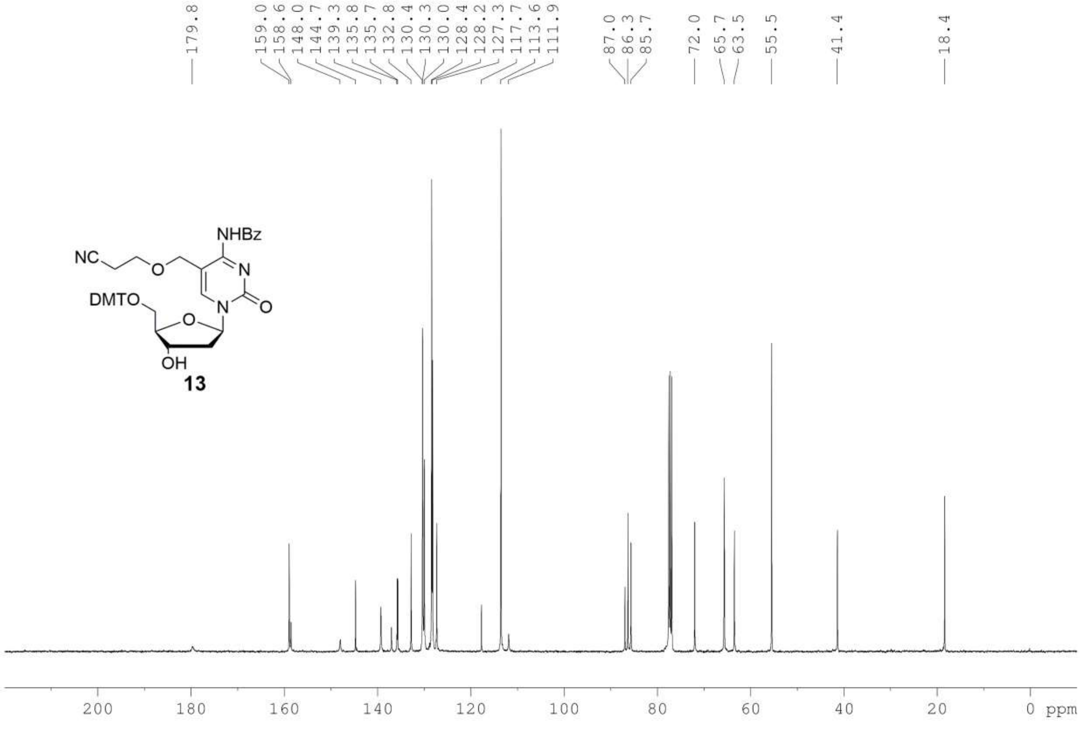

Figure S8. ^{13}C NMR spectrum of **13**

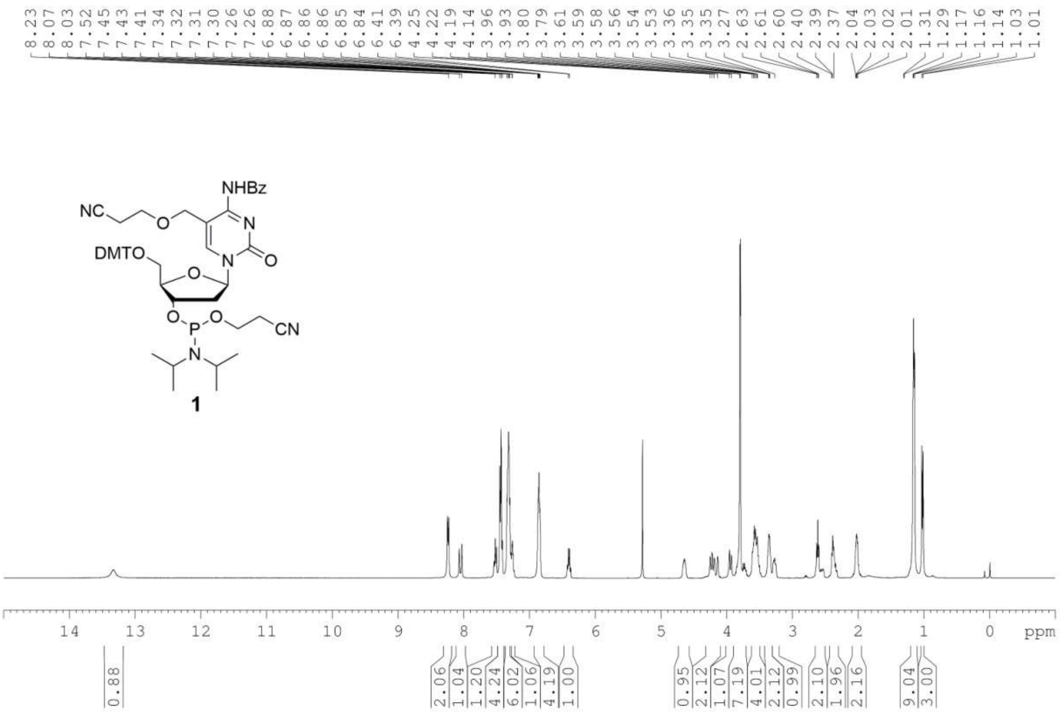

Figure S9. ^{1}H NMR spectrum of **1**

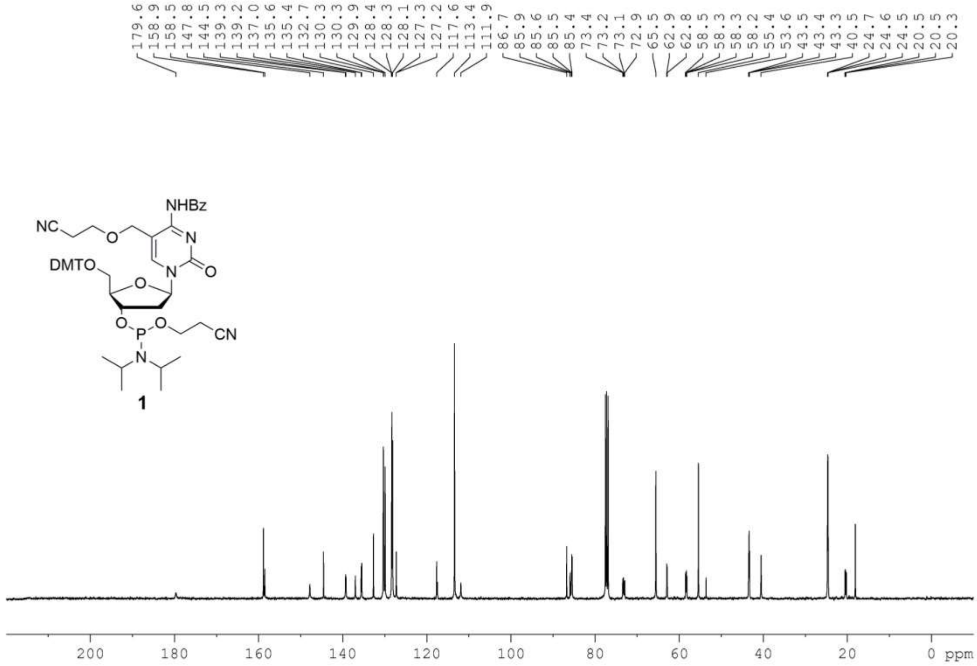

Figure S10. ^{13}C NMR spectrum of **1**

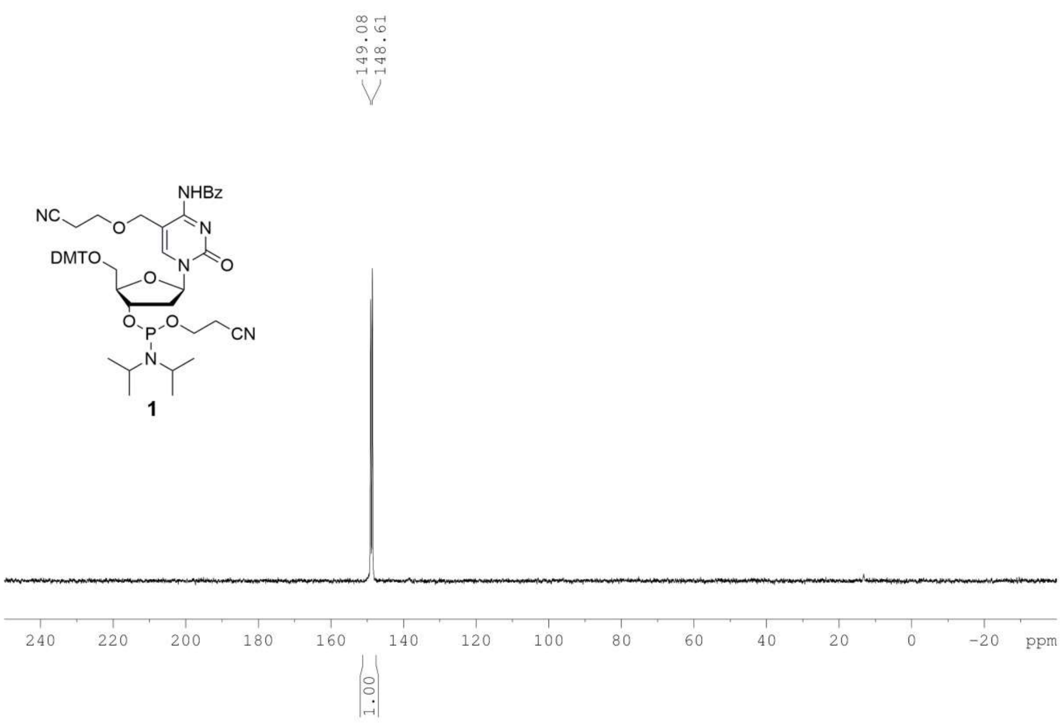

Figure S11. ^{31}P NMR spectrum of **1**

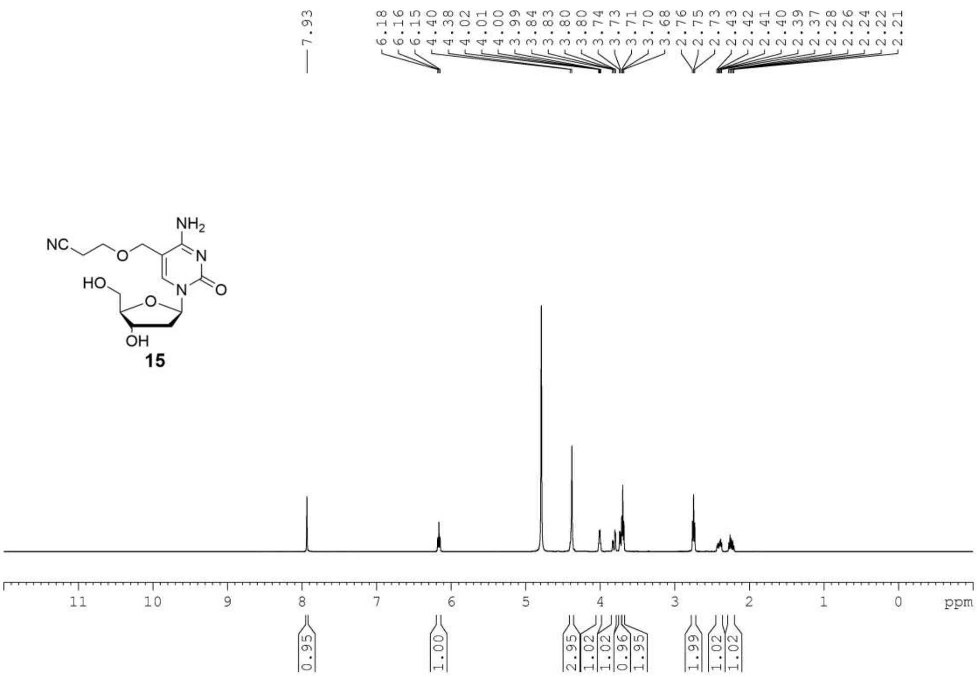

Figure S12. ^{1}H NMR spectrum of **15**

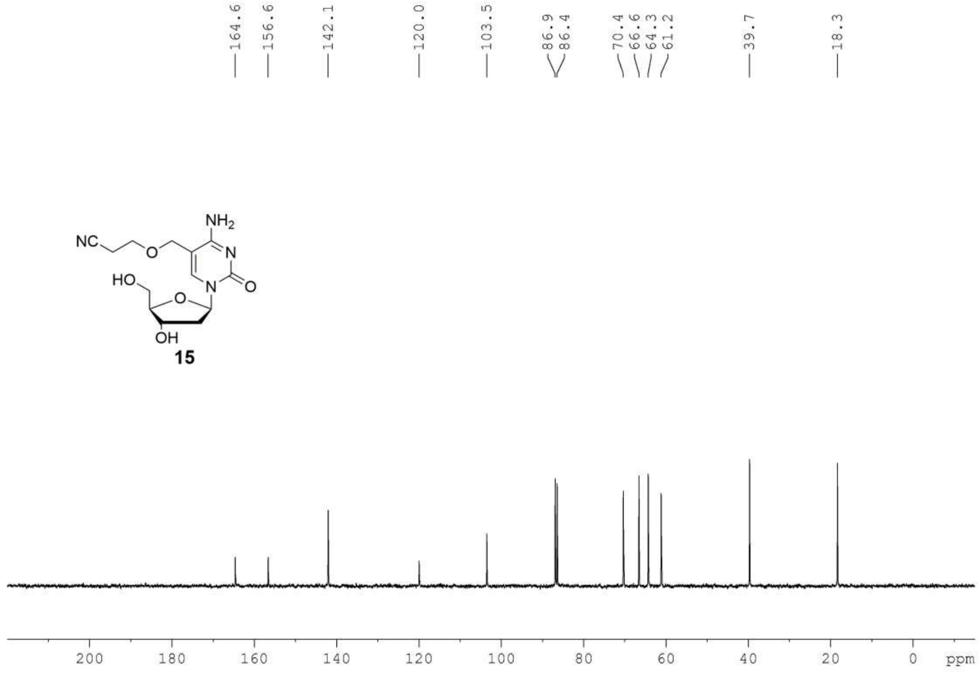

Figure S13. ^{13}C NMR spectrum of **15**

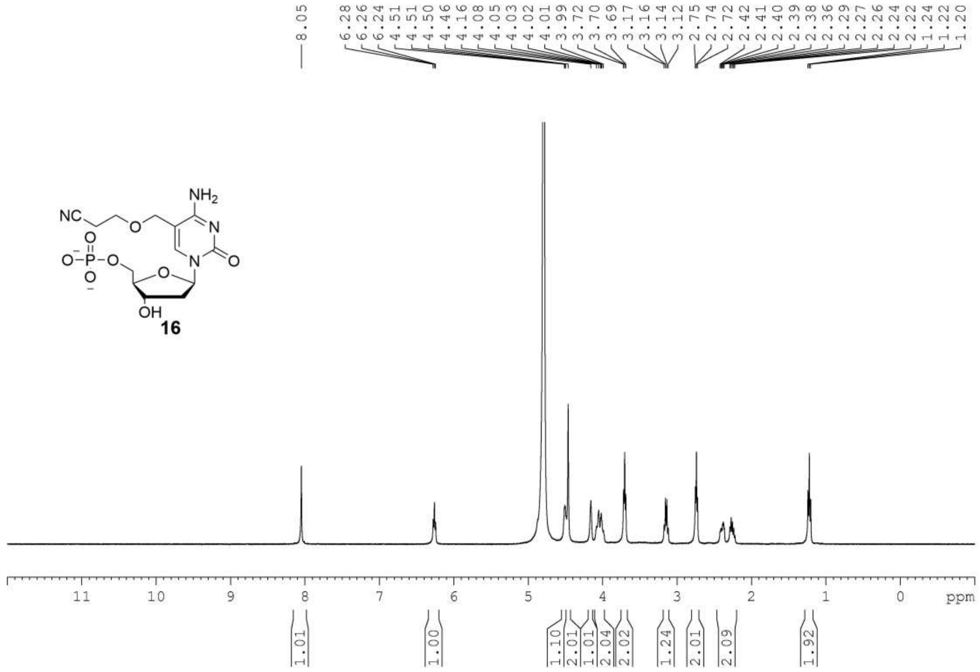

Figure S14. ^{1}H NMR spectrum of **16**

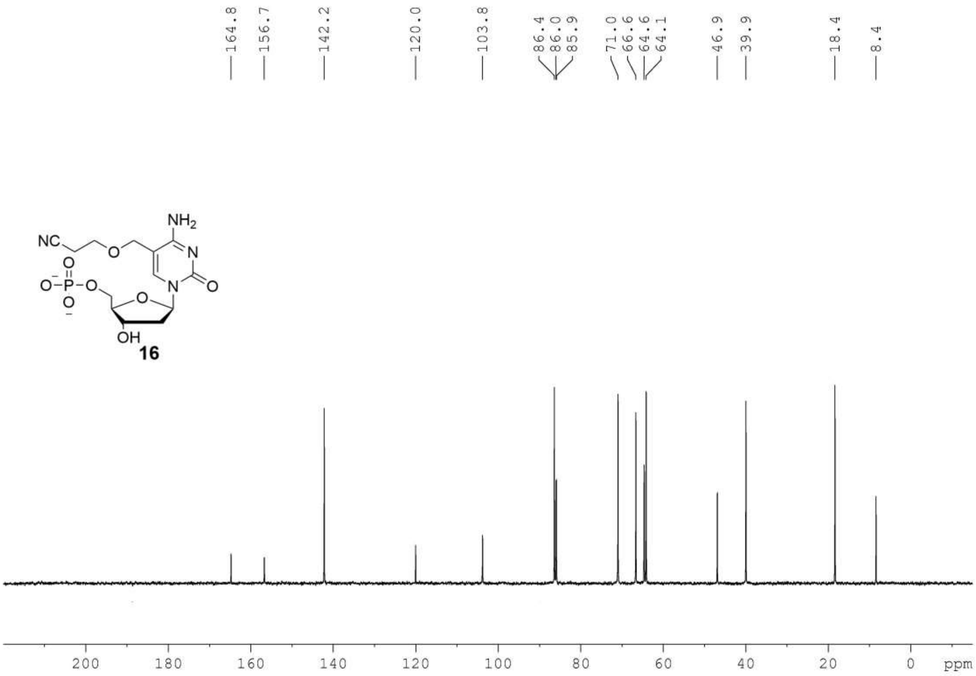

Figure S15. ^{13}C NMR spectrum of **16**

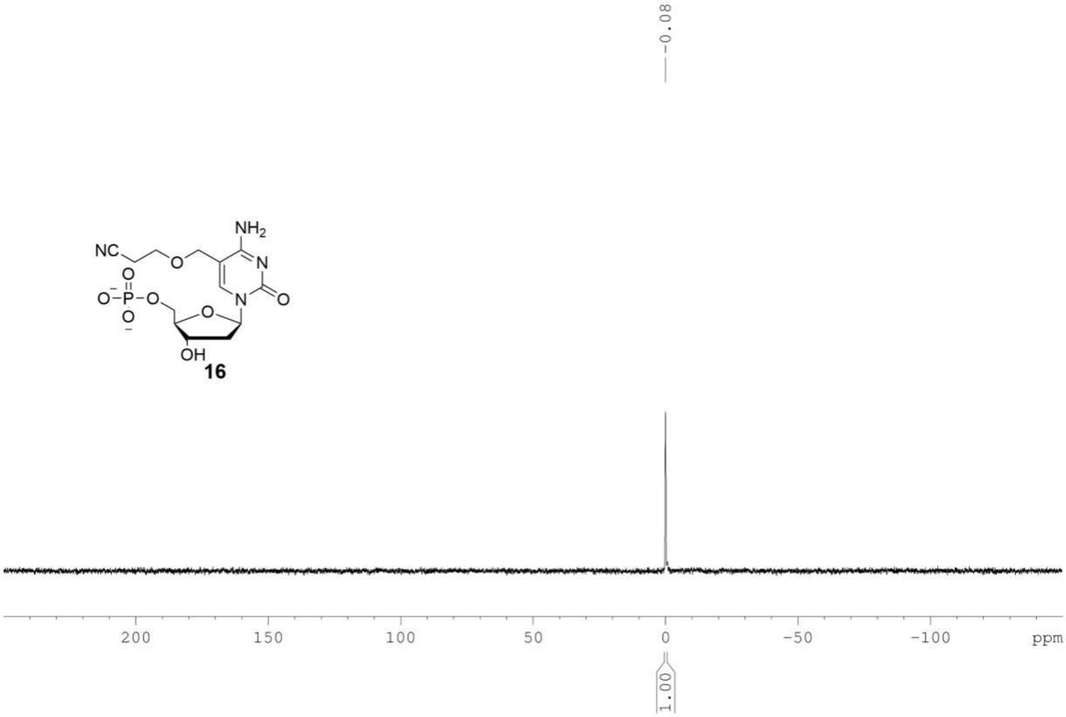

Figure S16. ^{31}P NMR spectrum of **16**

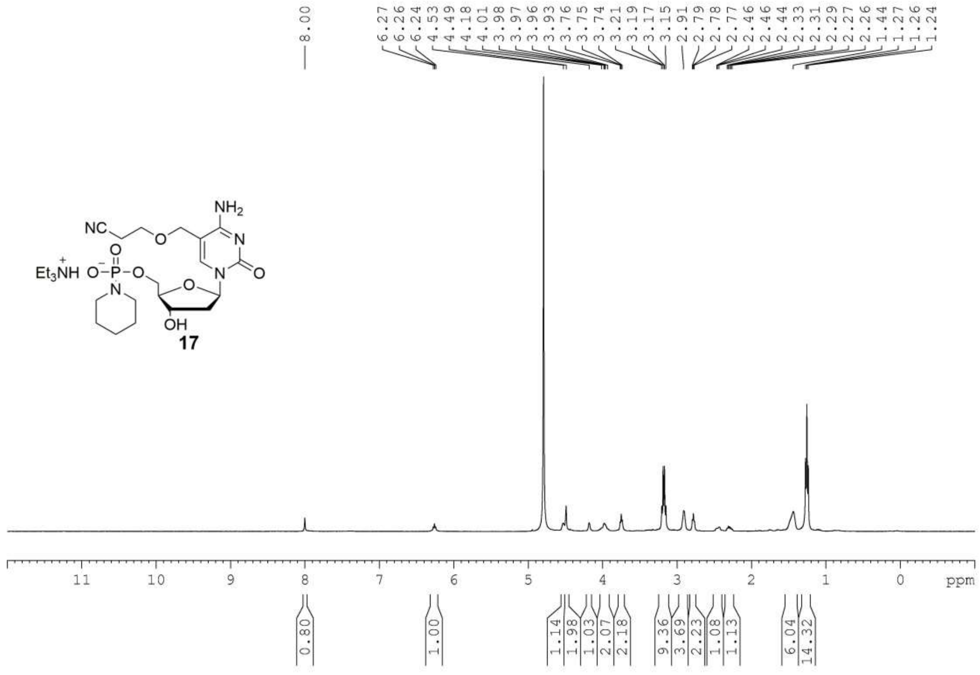

Figure S17. ^{1}H NMR spectrum of **17**

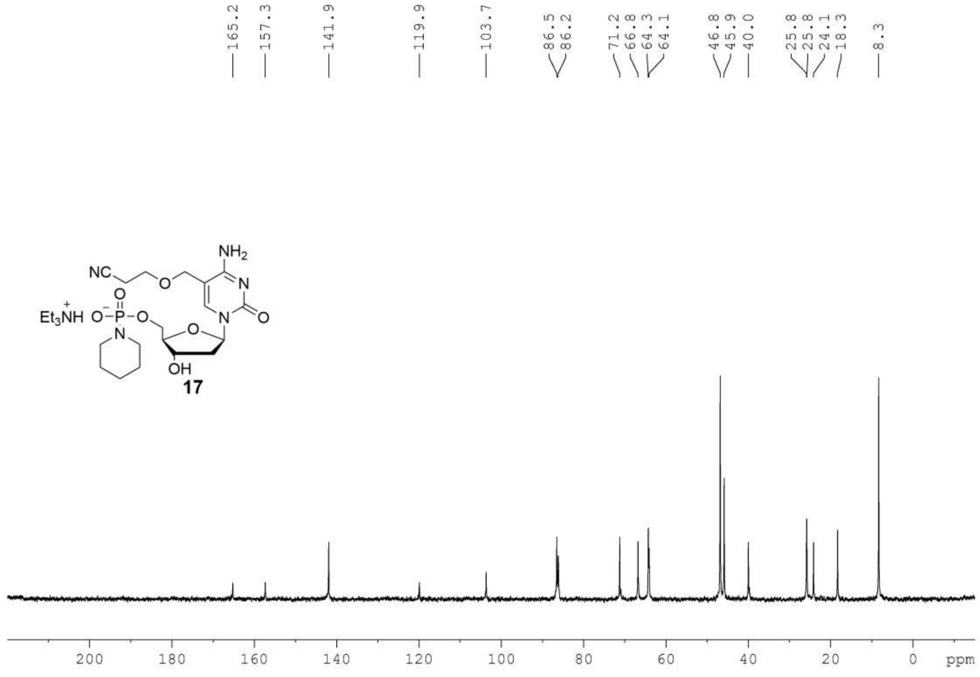

Figure S18. ^{13}C NMR spectrum of **17**

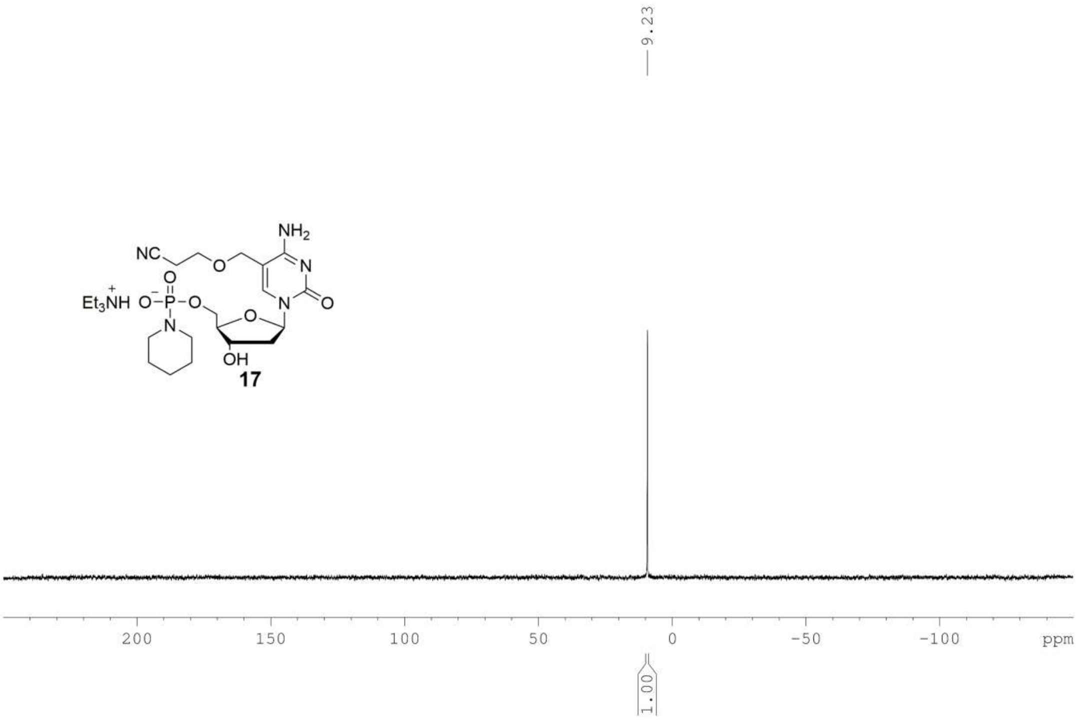

Figure S19. ^{31}P NMR spectrum of **17**

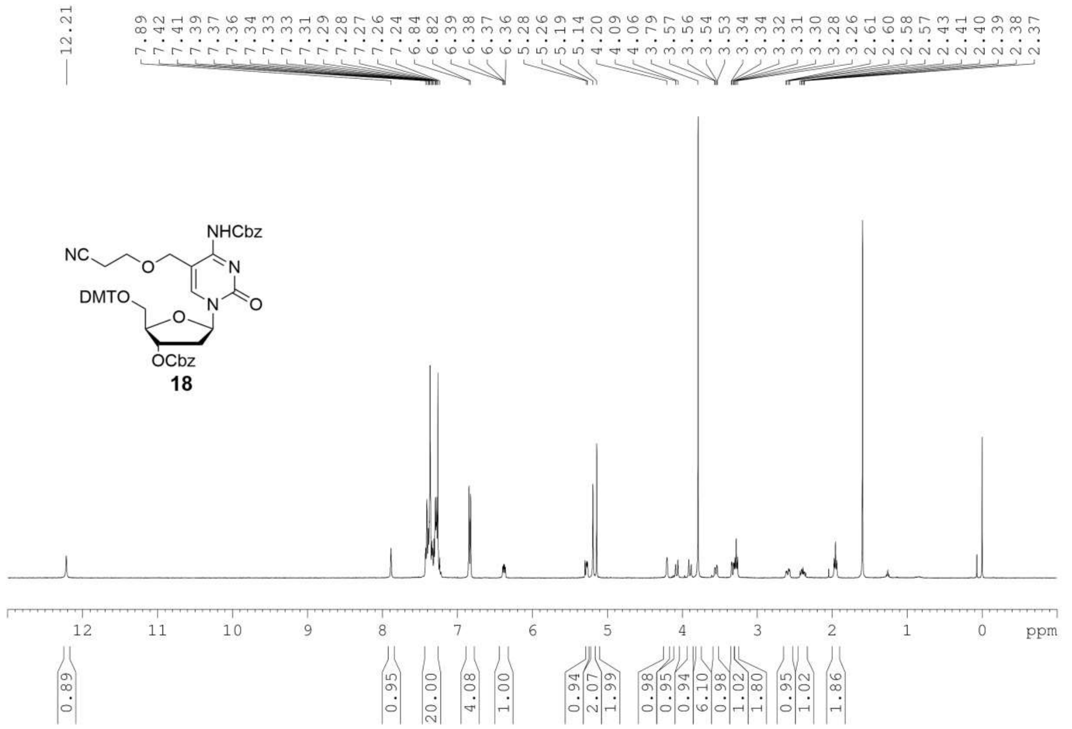

Figure S20. ^{1}H NMR spectrum of **18**

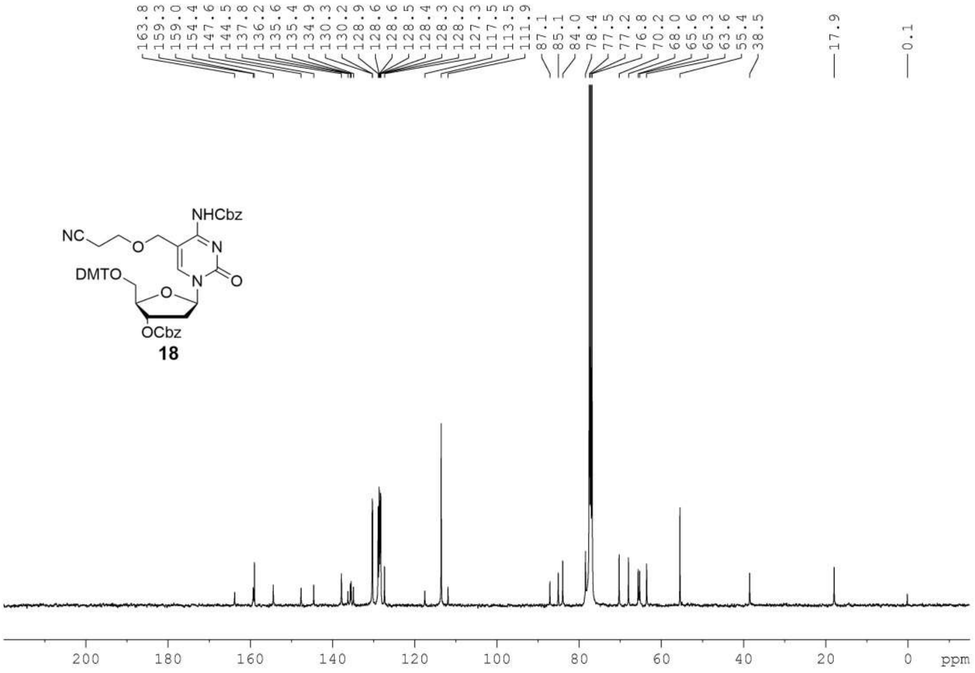

Figure S21. ^{31}C NMR spectrum of **18**

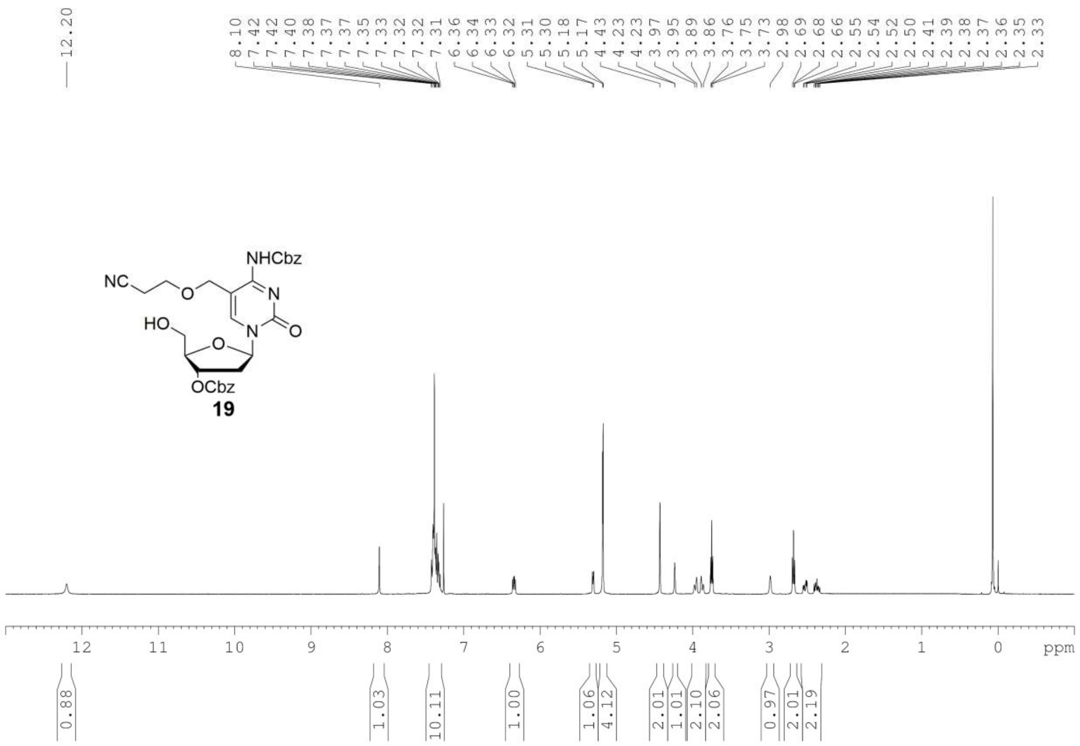

Figure S22. ^{1}H NMR spectrum of **19**

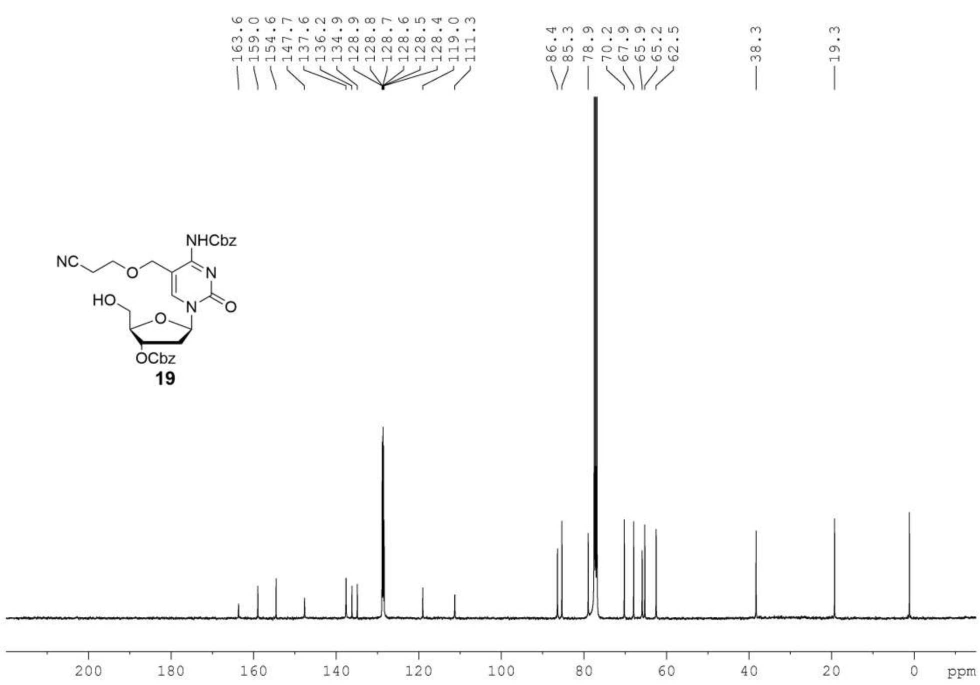

Figure S23. ^{13}C NMR spectrum of **19**

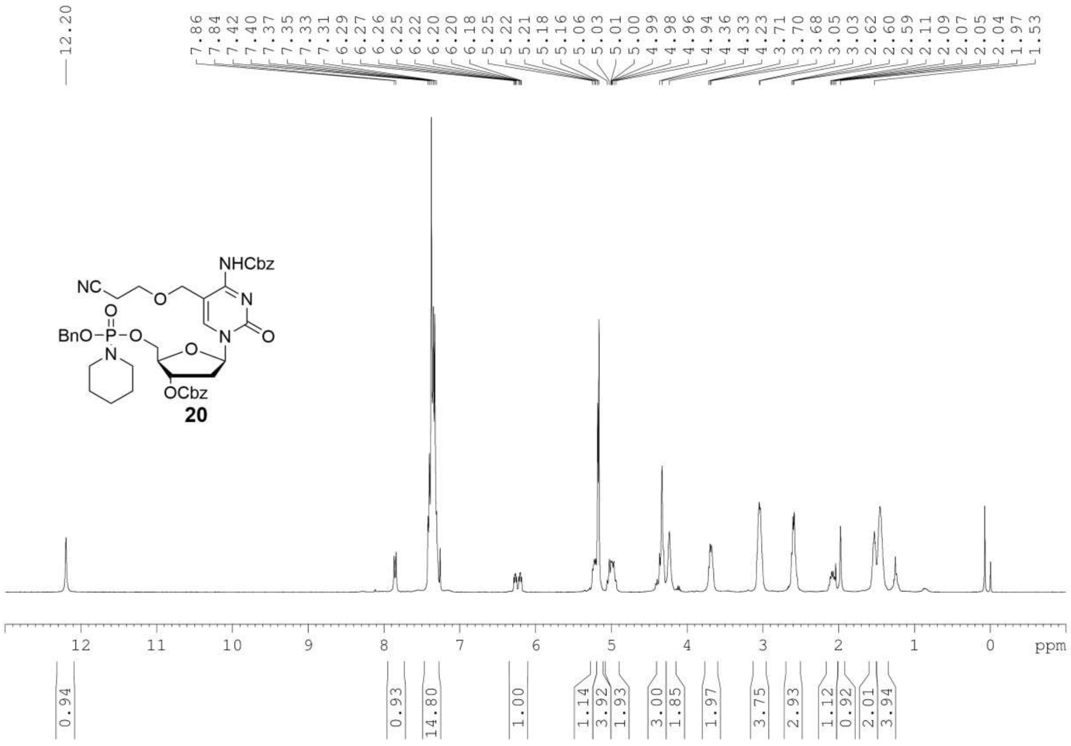

Figure S24. ^{1}H NMR spectrum of **20**

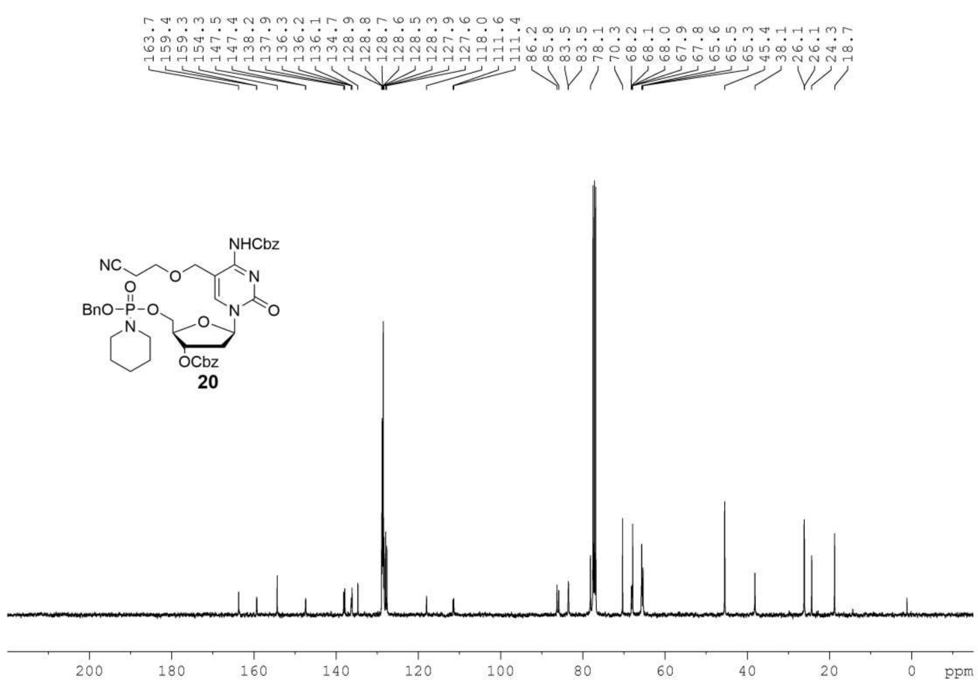

Figure S25. ^{13}C NMR spectrum of **20**

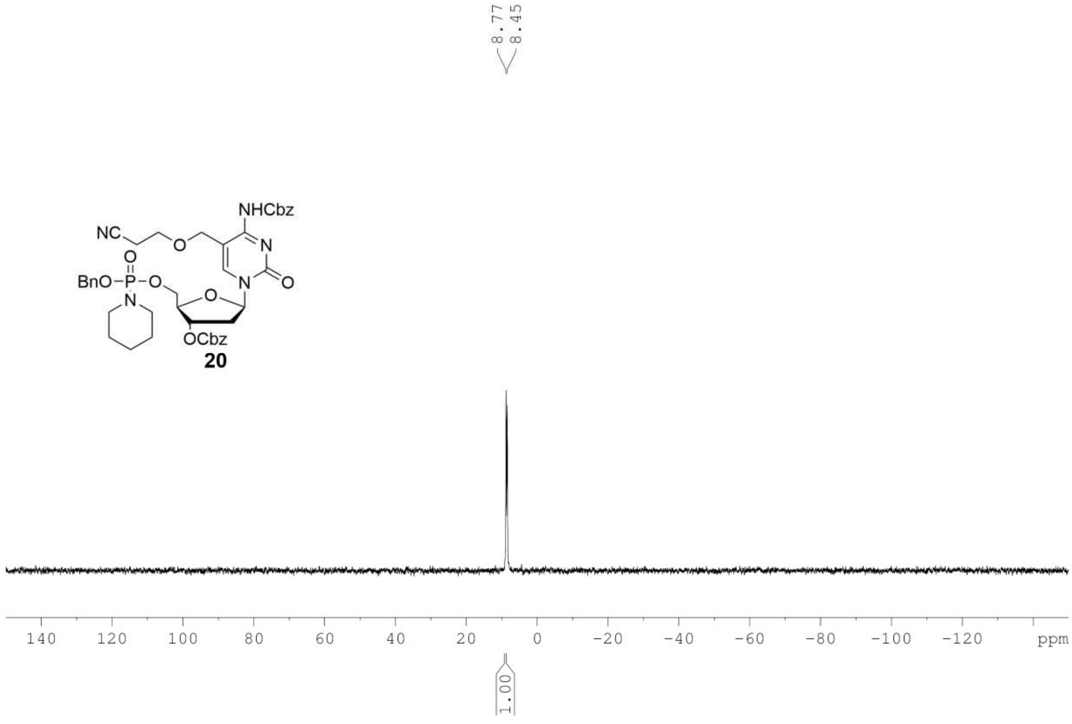

Figure S26. ^{31}P NMR spectrum of **20**

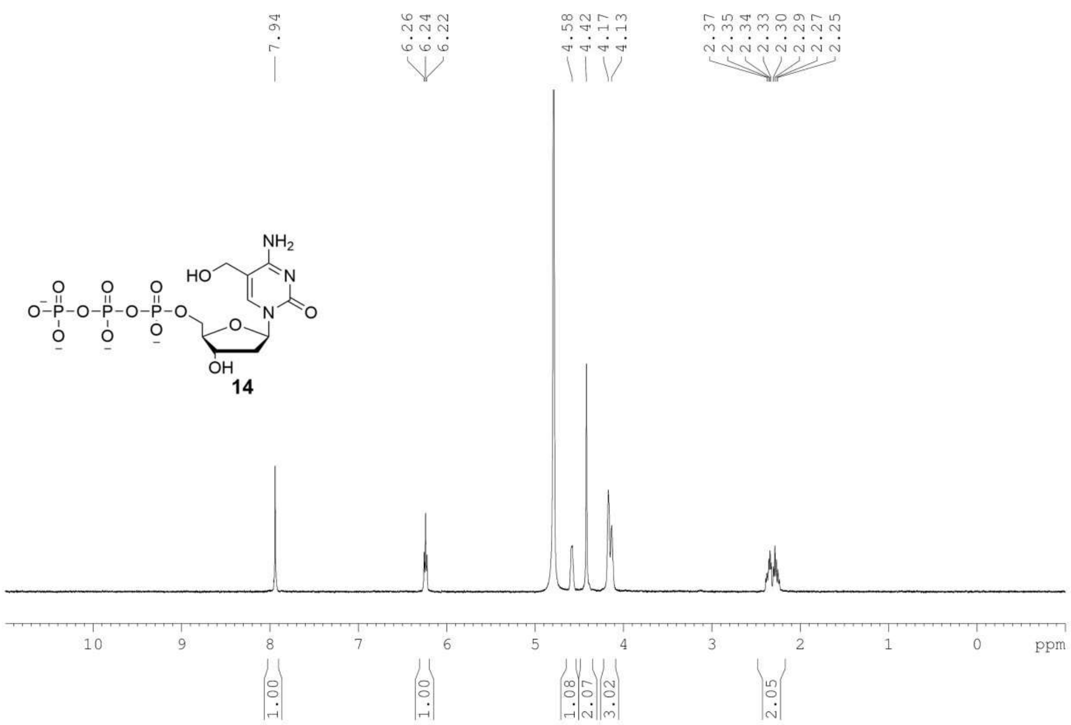

Figure S27. ^{1}H NMR spectrum of **14**

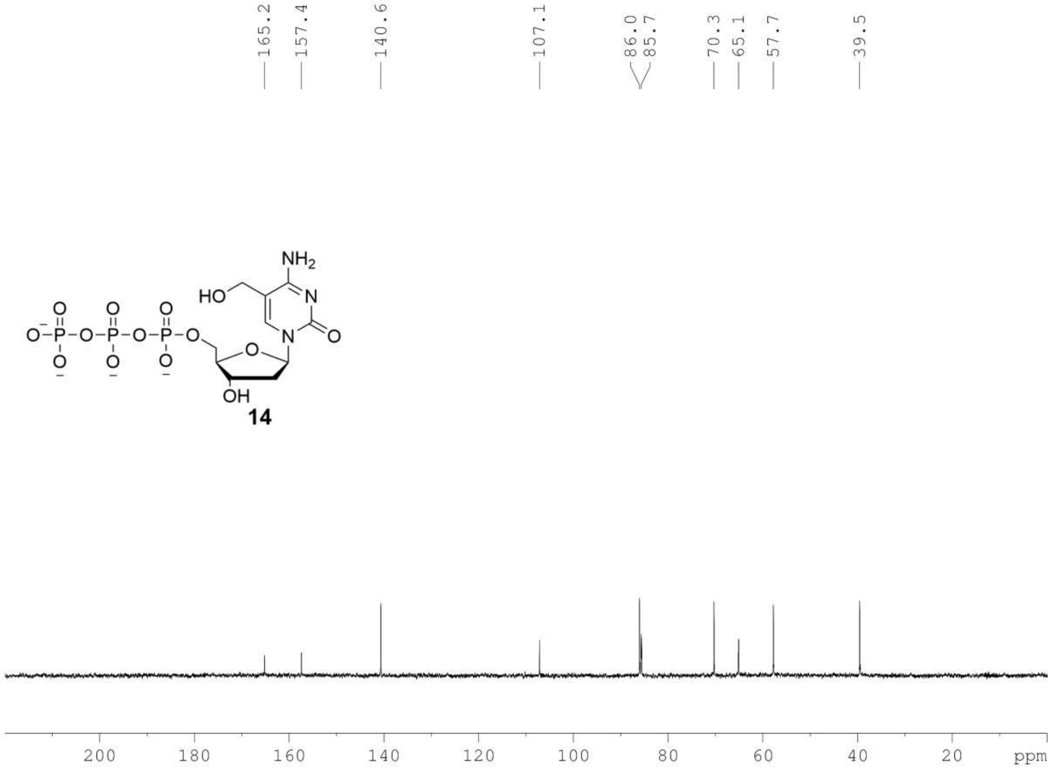

Figure S28. ^{13}C NMR spectrum of **14**

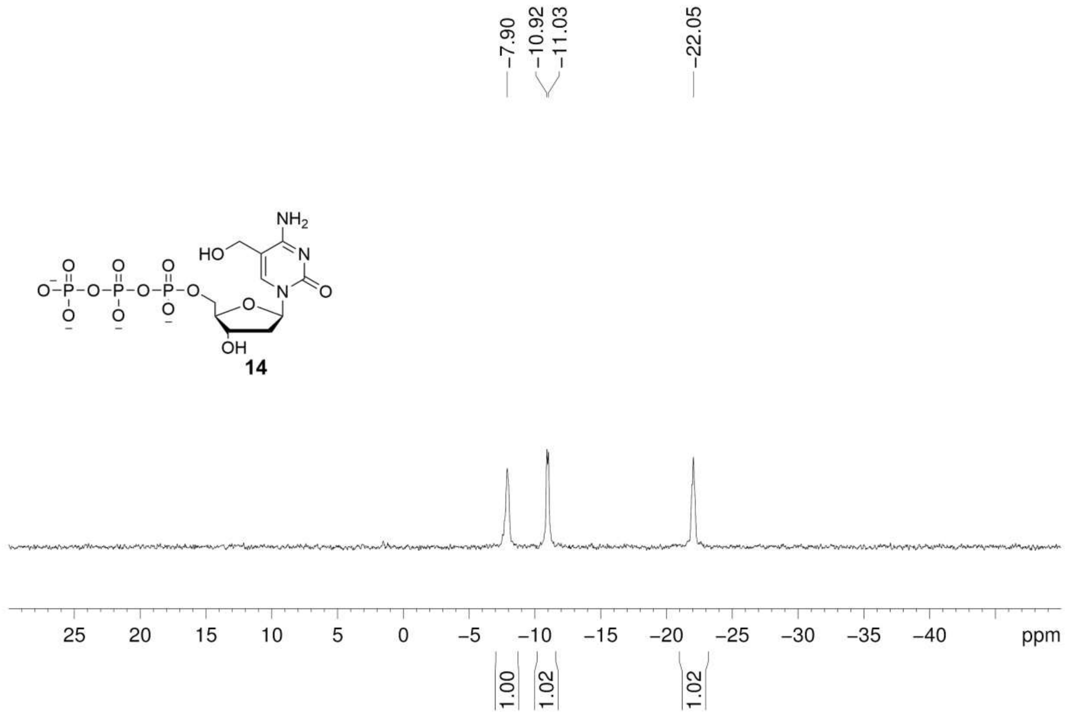

Figure S29. ^{31}P NMR spectrum of **14**
